# Selective autophagic receptor NbNBR1 prevents NbRFP1-mediated UPS-dependent degradation of βC1 to promote geminivirus infection

**DOI:** 10.1371/journal.ppat.1009956

**Published:** 2021-09-27

**Authors:** Tingting Zhou, Mingzhen Zhang, Pan Gong, Fangfang Li, Xueping Zhou

**Affiliations:** 1 State Key Laboratory of Rice Biology, Institute of Biotechnology, Zhejiang University, Hangzhou, Zhejiang, China; 2 State Key Laboratory for Biology of Plant Diseases and Insect Pests, Institute of Plant Protection, Chinese Academy of Agricultural Sciences, Beijing, China; The Ohio State University, UNITED STATES

## Abstract

Autophagy is an evolutionarily conserved, lysosomal/vacuolar degradation mechanism that targets cell organelles and macromolecules. Autophagy and autophagy-related genes have been studied for their antiviral and pro-viral roles in virus-infected plants. Here, we demonstrate the pro-viral role of a selective autophagic receptor NbNBR1 in geminivirus-infected *Nicotiana benthamiana* plants. The βC1 protein encoded by tomato yellow leaf curl China betasatellite (TYLCCNB) that is associated with tomato yellow leaf curl China virus (TYLCCNV) enhanced the expression level of *NbNBR1*. Then NbNBR1 interacted with βC1 to form cytoplasmic granules. Interaction of NbNBR1 with βC1 could prevent degradation of βC1 by the NbRFP1, an E3 ligase. Overexpression of NbNBR1 in *N*. *benthamiana* plants increased βC1 accumulation and promoted virus infection. In contrast, silencing or knocking out *NbNBR1* expression in *N*. *benthamiana* suppressed βC1 accumulation and inhibited virus infection. A single amino acid substitution in βC1 (βC1^K4A^) abolished its interaction with NbNBR1, leading to a reduced level of βC1^K4A^. The TYLCCNV/TYLCCNB^K4A^ mutant virus caused milder disease symptoms and accumulated much less viral genomic DNAs in the infected plants. Collectively, the results presented here show how a viral satellite-encoded protein hijacks host autophagic receptor NbNBR1 to form cytoplasmic granules to protect itself from NbRFP1-mediated degradation and facilitate viral infection.

## Author summary

We provide new evidence to show that βC1 encoded by tomato yellow leaf curl China betasatellite (TYLCCNB) that is associated with tomato yellow leaf curl China virus (TYLCCNV) can modulate plant defense against geminivirus infection. The βC1 protein could increase the expression level of *NbNBR1*, known as a selective autophagic receptor protein, and interact with it to form cytoplasmic granules *in planta*. Interaction between NbNBR1 and βC1 restrains interaction between NbRFP1, an E3 ligase, and βC1, thus to stabilize βC1 away from NbRFP1-mediated degradation. Hijacking of an autophagic receptor NbNBR1 by βC1 to benefit geminivirus infection has not been reported previously. This study demonstrates a distinct mechanism of a viral satellite-encoded protein exploits the autophagy protein NbNBR1 to the cytoplasmic granules, thus, to prevent the NbRFP1-mediated degradation and highlight an important role for NbNBR1 in geminivirus infection.

## Introduction

Autophagy, originated from a Greek word describing self-eating [1], is an evolutionary conserved degradation pathway that targets macromolecules, organelles and/or pathogens [2]. Three main types of autophagy have been reported in eukaryotes: macroautophagy (thereafter referred to as autophagy), microautophagy and chaperone-mediated autophagy [3]. Autophagosomes are double membrane-bound vesicles formed through a series of steps, and are important components involved in the autophagy pathway. These membrane-bound vesicles fuse to host cell vacuoles (yeast and plants) or lysosomes (mammals) to deliver cargos for proteolytic degradation and monomer recycling. More than 40 autophagy-related proteins (ATGs) have been identified in plants and function in autophagy induction, phagophore nucleation, autophagosomes expansion, and vacuolar membrane fusion [2]. Autophagy was previously considered as bulk and non-selective. In recent years, more and more reports have shown that autophagy operates in selective ways via selective autophagic cargo adaptors [4]. For example, cargo adaptor p62/Sequestosome-1 (p62/SQSTM1) and neighbor of BRCA1 gene 1 (NBR1) recognize and bind ubiquitinated protein complexes, while BCL2/adenovirus E1B 19 kDa-interacting protein 3 (BNIP3) targets endoplasmic reticulum and mitochondria for autophagosome degradation [5,6]. In a different report, Beclin1 (ATG6) has been shown as a new selective plant autophagy receptor that can mediate autophagic degradation and inhibit the replication and infection of turnip mosaic virus (TuMV) through interaction with viral RNA-dependent RNA polymerase NIb [7].

Based on the microarray study, Laura et al. found that many genes involved in autophagy pathway were up-regulated in tomato after infection by tomato yellow leaf curl Sardinia virus [8]. Many reports have demonstrated that autophagy can be activated upon virus infection to play important roles in plant resistance to virus infection [9–14]. For example, autophagy can regulate plant hypersensitive cell death (PCD) response to restrict tobacco mosaic virus (TMV) infection [15]. ATG8f, also an autophagy-associated protein, has been shown to interact with cotton leaf curl Multan virus (CLCuMuV) βC1 protein to suppress virus infection [16]. *Arabidopsis* NBR1 (AtNBR1) has been shown to directly target cauliflower mosaic virus (CaMV) capsid protein (CP) for degradation, while CaMV safeguards its CP via sequestering it into viral inclusions [17]. In addition, the NBR1-mediated selective autophagy could suppress TuMV infection via targeting viral RNA silencing suppressor HC-Pro [18]. To prevent this degradation, TuMV utilizes its own proteins to interfere the NBR1-mediated selective autophagy [18].

During plant and virus arm races, virus has evolved different strategies to combat host defenses against viral replication and/or infections, including antagonizing autophagy directly or hijacking autophagic components to facilitate viral replication and infection. The γb protein of barley stripe mosaic virus (BSMV) has been shown to disturb the autophagy-mediated degradation through competing with ATG8 for ATG7 [19]. Bamboo mosaic virus (BaMV) infection induces the expressions of *ATGs*, and among these *ATGs*, ATG8f has been shown to associate with chloroplast-derived vesicles that are ideal sites for viral RNA replication and protection of viral RNAs from host silencing machinery [20]. TuMV 6K2 protein was reported to induce *NBR1* expression, and TuMV could exploit the NBR1-ATG8f-mediated autophagy via an interaction between viral NIb and NBR1 to target viral replication complexes (VRCs) to tonoplasts for virus replication and virion formation [21].

Geminiviruses contain single-stranded circular DNA genomes and each genomic DNA encodes 4–6 classical viral proteins and many additional small viral proteins identified in a recent report [22]. Geminiviruses are transmitted by insect vectors and can infect many economically important crops, causing significant losses to agricultural industries worldwide [23]. The genus *Begomovirus* contains the largest member of species and is divided into the monopartite and bipartite begomoviruses, according to their genome components. Most monopartite begomoviruses are known to have betasatellites [24,25], which are circular single-stranded DNAs with about 1,350 nucleotides (nt). βC1 is a betasatellite-encoded protein and is a determinant of disease symptoms as well as a repressor of host defense [26]. For example, tomato yellow leaf curl China betasatellite (TYLCCNB)-encoded βC1 is reported to counteract plant defense responses including transcriptional gene silencing, post-transcriptional gene silencing, and mitogen-activated protein kinase-mediated plant immunity [27–29]. βC1 is also a key target of plant defense. Tomato (*Solanum lycopersicum*) SNF1-related kinase (SlSnRK1) has been shown to function defense against geminivirus infection through phosphorylation of viral βC1 [30]. *Nicotiana tabacum* E3 ligase, a RING-finger protein known as NtRFP1, has been shown to interact with and mediate βC1 degradation by the ubiquitin-26S proteasome system (UPS) [31].

In this study, we have identified a *N*. *benthamiana* selective autophagic receptor NbNBR1, a homologous of AtNBR1 in *Arabidopsis* and NtJoka2 in *N*. *tabacum*. TYLCCNB βC1 up-regulates the expression level of *NbNBR1*, and interacts with NbNBR1 to form cytoplasmic granules. NbNBR1 could prevent NbRFP1-mediated UPS-dependent degradation of βC1 to benefit geminivirus infection. Different from previous reports, our results indicate that a selective autophagy receptor can be exploited by a specific viral satellite-encoded protein. This new finding unveils a previously unknown mechanism modulating the roles of autophagy-related proteins in the arm race between plant and geminivirus.

## Results

### NbNBR1 interacts with βC1 to form cytoplasmic granules

**Fig 1:**
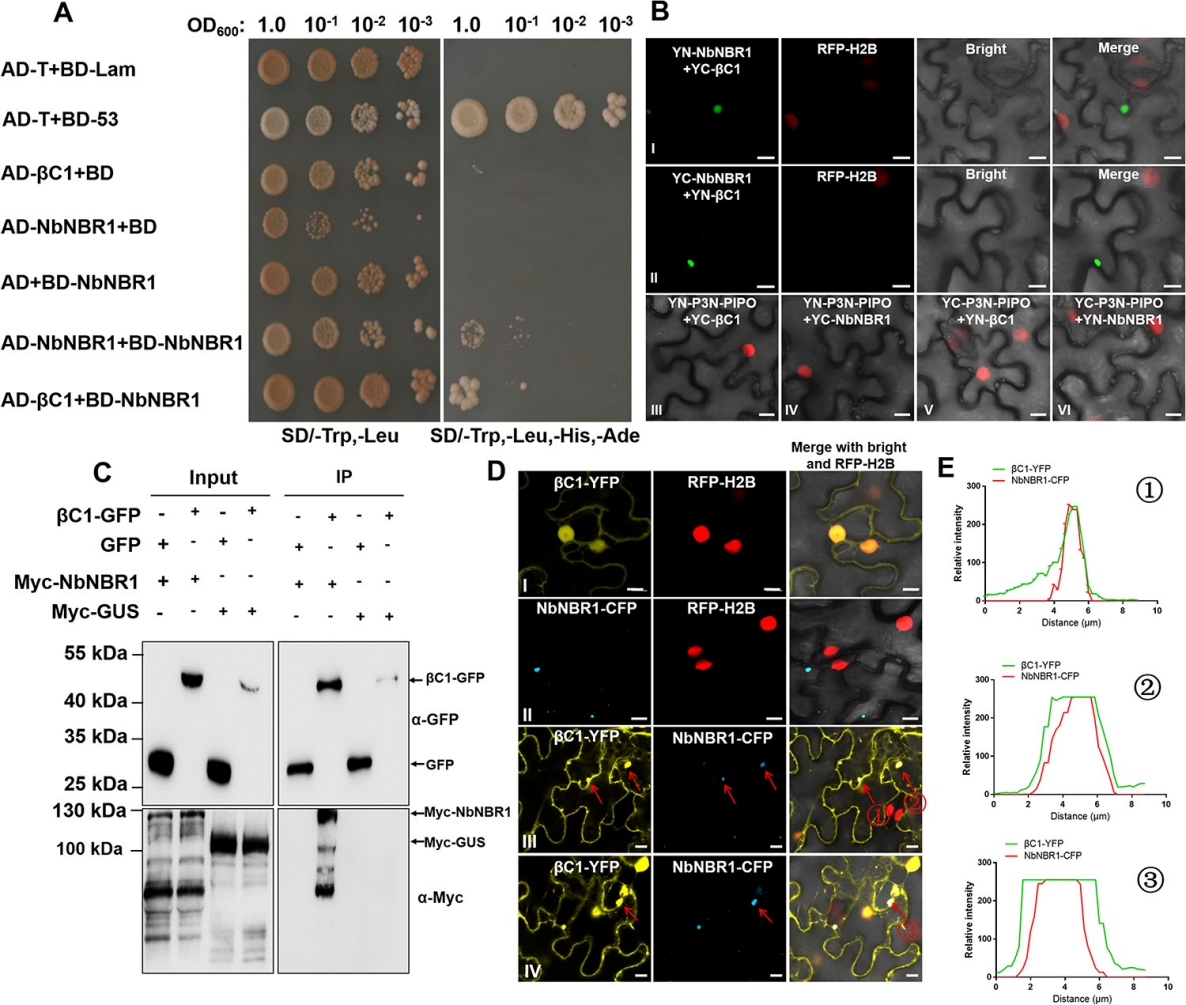
Determination of direct interaction between NbNBR1 and βC1. (A) Yeast two-hybrid (Y2H) assay was performed to determine the interaction between NbNBR1 and βC1. Y2H Gold yeast cells co-expressing AD-T+BD-53 or AD-T+BD-Lam were used as the positive and the negative controls, respectively. BD, binding domain of GAL4; AD, activation domain of GAL4. (B) BiFC assay was performed in the RFP-H2B transgenic *N*. *benthamiana* leaves. At 48 hpi, yellow fluorescence (green) was examined in the cells co-expressing the plasmids as indicated above. Bar = 10 μm. (C) Co-IP assay was performed to determine protein-protein interactions. βC1-GFP, GFP, Myc-NBR1, and Myc-GUS were co-expressed, as indicated, in *N*. *benthamiana* leaves through agroinfiltration. Blots with input samples (Input) or IP samples (IP) were probed with anti-GFP or anti-Myc antibodies. Arrows indicate the expected protein bands. (D) Subcellular localization patterns of NbNBR1-CFP and βC1-YFP in RFP-H2B transgenic *N*. *benthamiana* leaf cells. (I) means the expression of βC1-YFP alone in plant cell. (II) means the expression of NbNBR1-CFP alone in plant cell. (III-IV) means co-expression of βC1-YFP and NbNBR1-CFP in plant cell. YFP, RFP, and CFP fluorescence were captured under a confocal microscope. Arrows show the granules from overlapping of NbNBR1-CFP and βC1-YFP. Bar = 10 μm. The integrity of fused proteins in Fig 1D was confirmed by Western blot (S1B Fig). (E) Fluorescence intensity of cytoplasmic granules at Fig 1D (III and IV) was quantified by ZEN 3.1 (Carl Zeiss ZEN system). The overlapping fluorescence spectra of βC1-YFP and NbNBR1-CFP from the red arrow marked region of Fig 1D (III and IV) showed their co-localization.

To investigate the role of autophagy in the infection of geminivirus associated with a betasatellite, we used βC1 as a bait to screen βC1-interacting autophagy-related proteins via Y2H assay. The result showed that the yeast cells transformed with AD-βC1+BD-NbNBR1 grew on the selective medium, while the yeast cells transformed with AD-βC1+BD or AD+BD-NbNBR1 did not (Fig 1A). To further validate this interaction, we performed bimolecular fluorescence complementation (BiFC) assay in the leaves of the RFP-H2B transgenic *N*. *benthamiana* plants. The N-terminal half or the C-terminal half of YFP was fused to the C-terminus of NbNBR1 or βC1 to generate YN-NbNBR1, YC-NbNBR1, YN-βC1 and YC-βC1, respectively. Co-expression YN-NbNBR1+YC-βC1 or YC-NbNBR1+YN-βC1 in the RFP-H2B transgenic *N*. *benthamiana* leaves resulted in the formation of yellow fluorescent cytoplasmic granules (green) by 48 hours post infiltration (hpi), indicating a positive interaction between these two proteins. We also used TuMV P3N-PIPO, a viral movement protein, as a negative control. When YN-P3N-PIPO+YC-βC1, YN-P3N-PIPO+YC-NbNBR1, YC-P3N-PIPO+YN-βC1 or YC-P3N-PIPO+YN-NbNBR1 were co-expressed in leaves, no yellow fluorescent granules were observed (Fig 1B). In order to know if such interaction also happens in other sub-cellular compartments, we analyzed the co-localization of some reported markers of sub-cellular compartments and the NbNBR1-βC1 interaction complex. As shown in S1A Fig, we found that the interaction complex of NbNBR1-βC1 did not localize in chloroplast, Golgi, peroxisome or endosome, but it co-localized with the endoplamic reticulum (ER) marker. βC1-GFP, Myc-NbNBR1, GFP, and Myc-GUS were then transiently co-expressed for co-immunoprecipitation (Co-IP) assay using GFP-Trap_MA magnetic agarose beads (ChromoTek). As shown in Fig 1C, Myc-NbNBR1 rather than Myc-GUS was specifically immunoprecipitated by βC1-GFP, but not by GFP using anti-GFP and anti-Myc antibodies, confirming the presence of the NbNBR1-βC1 complex *in planta*.

Previously, our laboratory has reported that βC1 accumulates primarily in the cell nucleus and cytoplasm [32]. To investigate whether the interaction between NbNBR1 and βC1 could change the subcellular localization pattern of βC1, we expressed βC1-YFP and NbNBR1-CFP, individually or together, in the RFP-H2B transgenic *N*. *benthamiana* leaves. At 48 hpi, βC1-YFP expressed alone was observed in both nucleus and cytoplasm, while NbNBR1-CFP expressed alone was observed as small granules in the cytoplasm. In the cells co-expressing βC1-YFP and NbNBR1-CFP, some βC1-YFP was found to co-localize with NbNBR1-CFP to form cytoplasmic granules (Fig 1D). The overlapping fluorescence signal and spectra of βC1-YFP and NbNBR1-CFP (Fig 1D and 1E) further confirmed that βC1 and NbNBR1 were co-localized in the cytoplasmic granules.

### Silencing and knocking out of *NbNBR1* expression inhibits TYLCCNV/TYLCCNB infection

**Fig 2:**
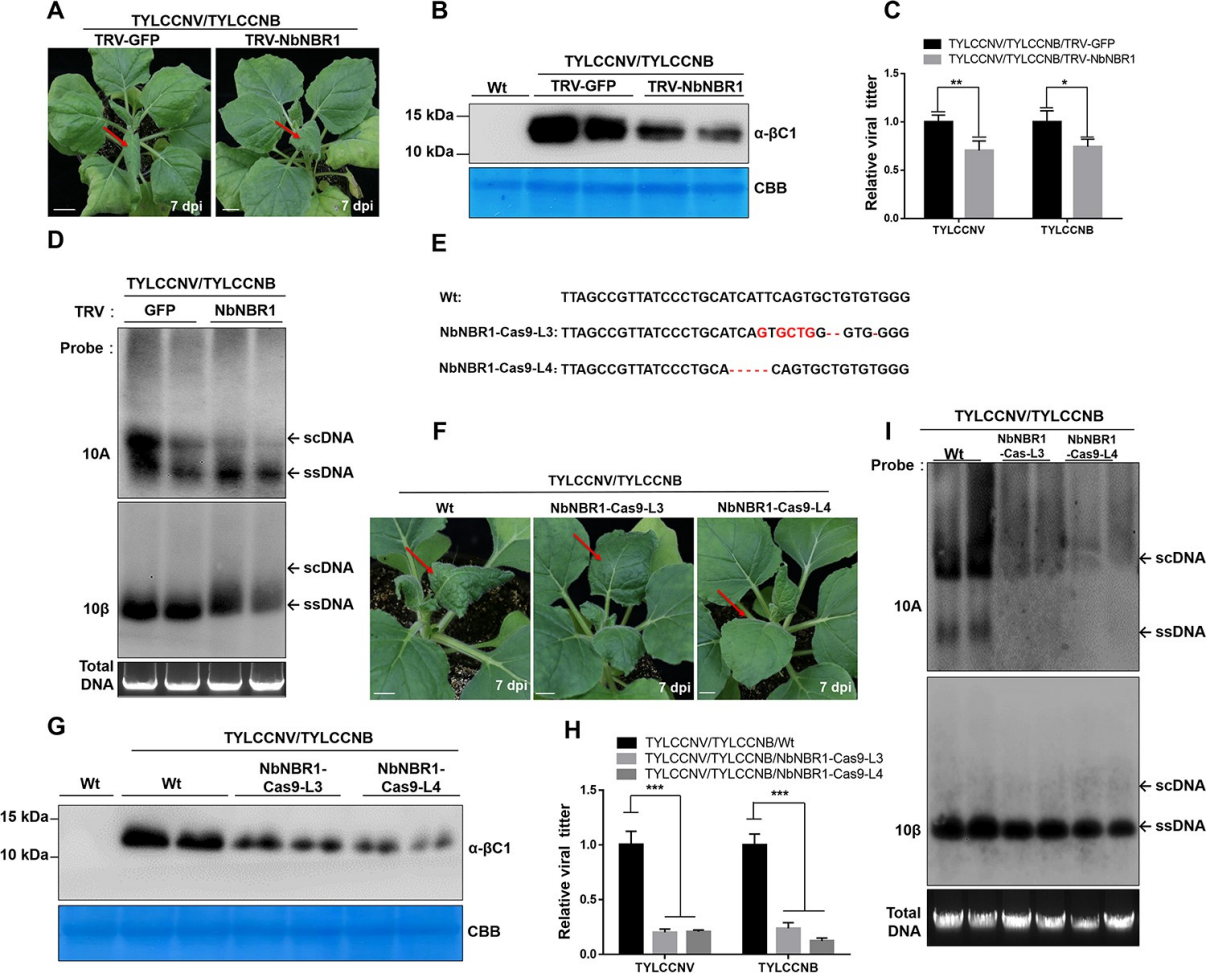
Knockdown or knockout of *NbNBR1* inhibits TYLCCNV/TYLCCNB infection in *N*. *benthamiana* plants. (A) Phenotypes of the TYLCCNV/TYLCCNB-inoculated non-silenced and *NbNBR1*-silenced *N*. *benthamiana* plants at 7 dpi. Red arrows indicate the leaves showing leaf curling symptoms. Bar = 2 cm. Wt: wild type *N*. *benthamiana* plants. (B) Western blot analyses of βC1 accumulation using anti-βC1 antibodies in the TYLCCNV/TYLCCNB-inoculated non-silenced or *NbNBR1*-silenced *N*. *benthamiana* leaves at 3 dpi. The CBB-stained Rubisco large subunit gels were used to show equal sample loadings. (C) Relative viral accumulations of TYLCCNV and TYLCCNB DNA in the plants shown in (A) was detected by qPCR. 25S rRNA was used as an internal control, and values represent the mean ± standard deviation (SD) (n = 9). Double and single asterisks indicate significant statistical differences (**p<0.01 and *p<0.05, Student’s *t* test) between two treatments. (D) Southern blot analyses of the viral accumulation in the systemic leaves on the *NbNBR1*-silenced TYLCCNV/TYLCCNB infected plants at 14 dpi. The Gelstain-stained agarose gel was used to show equal sample loadings, viral single-stranded DNAs (ssDNA) and supercoiled DNA (scDNA) are indicated with arrows. (E) DNA sequencing and sequence alignment results confirmed the mutations and deletions in the *NbNBR1* gene sequence. These nucleotide deletions cause frame shifts in the coding region of *NbNBR1*. The T1 NbNBR1-Cas9 Line3 (NbNBR1-Cas9-L3) and NbNBR1-Cas9 Line4 (NbNBR1-Cas9-L4) plants were used for sequencing analyses. (F) Phenotypes of the TYLCCNV/TYLCCNB-inoculated Wt and *NbNBR1*-knockout *N*. *benthamiana* plants at 7 dpi, and red arrows indicate the leaves showing leaf curling symptoms. Bar = 2 cm. (G) Western blot assay was used to determine βC1 accumulation using anti-βC1 antibodies in the infiltrated leaves at 3 dpi (F), and the CBB-stained Rubisco large subunit gels were used to show equal sample loading. (H) qPCR analyses of TYLCCNV and TYLCCNB DNA accumulations in the systemic leaves of (F). 25S rRNA was used as an internal control, and the values presented are the means ± SD (n = 9). Triple asterisks indicate a significant statistical difference between two treatments (***p<0.001, Student’s *t* test). (I) Southern blot analyses of viral accumulation of TYLCCNV/TYLCCNB infected plants as indicated in (F) at 14 dpi. The Gelstain-stained agarose gel was used to show equal sample loadings, and viral ssDNA and scDNA are indicated with arrows.

To investigate the biological function of βC1 and NbNBR1 interaction, we silenced *NbNBR1* expression in *N*. *benthamiana* plants using a tobacco rattle virus (TRV)-based VIGS vector. The result showed that at 7 days post infiltration (dpi), the *NbNBR1*-silenced *N*. *benthamiana* plants (TRV-NbNBR1) did not show clear growth defects compared with the non-silenced control plants (TRV-GFP) (S2A Fig). Quantitative RT-PCR (qRT-PCR) analyses of *NbNBR1* expression showed that the expression level of *NbNBR1* in the *NbNBR1*-silenced *N*. *benthamiana* plants was decreased by 50% compared with the non-silenced plants (S2B Fig). We then inoculated the upper expanding young leaves of these plants with TYLCCNV/TYLCCNB. Seven days later, the non-silenced TYLCCNV/TYLCCNB-inoculated plants displayed typical leaf curling symptoms, while the *NbNBR1*-silenced TYLCCNV/TYLCCNB-inoculated plants showed a delayed and milder leaf curling symptom (Fig 2A). Western blot showed that the *NbNBR1*-silenced TYLCCNV/TYLCCNB-inoculated plants accumulated much less βC1 in the inoculated leaves at 3 dpi (Fig 2B). qPCR analyses at 7 dpi and Southern blot analyses at 14 dpi of viral DNA accumulations in the systemic leaves of plants showed less viral DNAs accumulated in the *NbNBR1*-silenced plants than the non-silenced plants (Fig 2C and 2D).

To further confirm the function of NbNBR1 described above, we generated two *NbNBR1*-knockout *N*. *benthamiana* lines using CRISPR/Cas9-based technology. The T1 NbNBR1-Cas9 Line3 and NbNBR1-Cas9 Line4 (referred to as NbNBR1-Cas9-L3 and NbNBR1-Cas9-L4, respectively) plants showed similar growth phenotypes as the Wt plants (S2C Fig). DNA sequencing results showed that the NbNBR1-Cas9-L3 line carried five altered and three deleted nts at the cleavage site, while the NbNBR1-Cas9-L4 line carried five deleted nts at the cleavage cite (Fig 2E). After inoculation of these plants with TYLCCNV/TYLCCNB through agroinfiltration, milder leaf curling symptoms appeared on *NbNBR1*-knockout *N*. *benthamiana* plants at 7 dpi compared to those in the Wt plant (Fig 2F). Western blot showed less βC1 accumulations in the virus-inoculated leaves of the NbNBR1-Cas9-L3 and NbNBR1-Cas9-L4 plants compared to those in the Wt plants at 3 dpi (Fig 2G). qPCR analyses at 7 dpi and Southern blot analyses at 14 dpi of viral DNA accumulations in the systemic leaves of plants indicated in Fig 2F, showed less viral DNAs accumulated in the *NbNBR1*-knouck out plants than the Wt plants (Fig 2H and 2I). Furthermore, to investigate whether NbNBR1 could affect the βC1-mediated disease symptom formation and virus accumulation, we inoculated the *NbNBR1*-silenced and non-silenced *N*. *benthamiana* plants with potato virus X (PVX) or a PVX-based vector expressing βC1 (PVX-βC1). By 7 dpi, PVX caused similar mosaic symptoms in both *NbNBR1*-silenced and non-silenced plants, while PVX-βC1 caused a delayed and milder leaf curling symptom in the *NbNBR1*-silenced plants compared to the non-silenced control plants (S2D Fig). Western blot analyses using anti-βC1 antibodies showed that the accumulation levels of βC1 in the *NbNBR1*-silenced PVX-βC1-inoculated plants were clearly reduced (S2E Fig). We also infiltrated NbNBR1-Cas9-L3 and NbNBR1-Cas9-L4 plants with PVX or PVX-βC1. Loss function of *NbNBR1* showed no influence on PVX-mediated symptom and PVX CP accumulation (S3A and S3B Fig), while less βC1 accumulation and weakened symptom were found on *NbNBR1-*knock out plants when infiltrated with PVX-βC1 (S3C and S3D Fig). These results indicated that NbNBR1 is important for the formation of βC1-induced symptoms.

### Transient overexpression of NbNBR1 increases βC1 accumulation to benefit TYLCCNV/TYLCCNB infection

**Fig 3:**
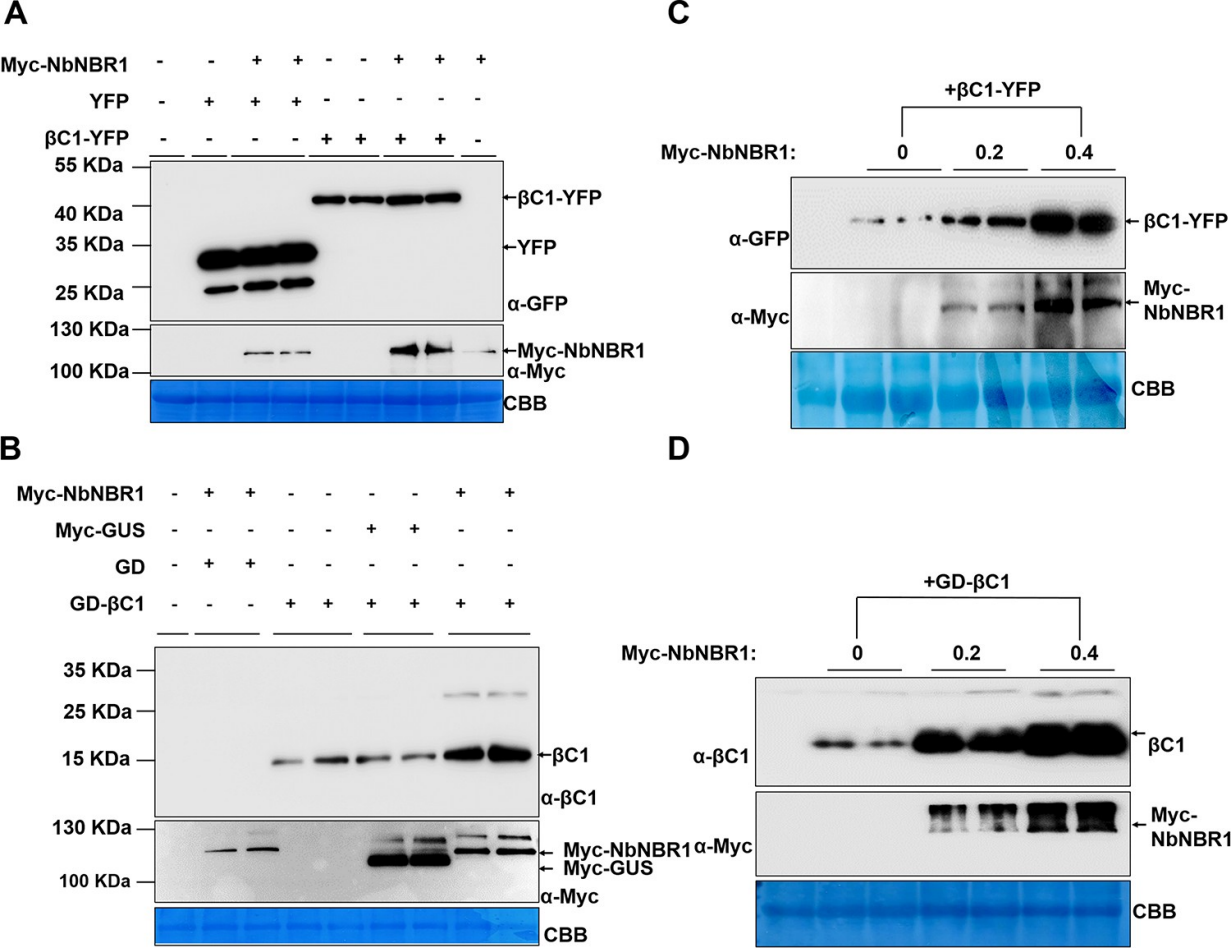
Co-expression of Myc-NbNBR1 increases βC1 accumulation. (A) Myc-NbNBR1, YFP, and βC1-YFP were transiently co-expressed, as indicated, in *N*. *benthamiana* leaves followed by Western blot assays using anti-GFP or anti-Myc antibodies. (B) Myc-NbNBR1, Myc-GUS, GD, and GD-βC1 were transiently co-expressed, as indicated, followed by Western blot assays. (C) *Agrobacterium tumefaciens* culture carrying Myc-NbNBR1 (OD_600_ = 0, 0.2 or 0.4) was mixed with *A*. *tumefaciens* cultures carrying βC1-YFP, and then infiltrated into *N*. *benthamiana* leaves followed by Western blot assays. (D) *A*. *tumefaciens* culture carrying Myc-NbNBR1 (OD_600_ = 0, 0.2 or 0.4) was mixed with *A*. *tumefaciens* cultures carrying GD-βC1, and then infiltrated into *N*. *benthamiana* leaves followed by Western blot assays. The CBB-stained Rubisco large subunit gels were used to show equal sample loadings (A-D). Each experiment was repeated three times with similar results. Arrows indicate the corresponding protein bands as indicated.

We transiently co-expressed Myc-NbNBR1 and βC1-YFP in *N*. *benthamiana* leaves through agroinfiltration to analyze the effect of NbNBR1 on βC1 accumulation. Western blot and end point qPCR results showed that co-expression of Myc-NbNBR1 and βC1-YFP increased the accumulation of βC1-YFP protein, but not βC1-YFP mRNA (Figs 3A and S4A). Furthermore, to avoid the potential effect of YFP tag, we transiently co-expressed Myc-NbNBR1, Myc-GUS, GD-βC1, and GD, in *N*. *benthamiana* leaves through agroinfiltration, and then analyzed these leaves for the accumulation of GD-βC1 through Western blot assay using anti-Myc and anti-βC1 antibodies. The results showed that transient co-expression of Myc-NbNBR1 and GD-βC1 did increase the accumulation of GD-βC1 (Fig 3B). When the concentration of *Agrobacterium tumefaciens* culture carrying Myc-NbNBR1 was increased from OD_600_ = 0.2 to 0.4 and mixed with an *A*. *tumefaciens* culture carrying βC1-YFP or GD-βC1 prior to agroinfiltration, the accumulation levels of βC1-YFP and GD-βC1 were also significantly increased (Fig 3C and 3D). Besides, we also conducted BiFC assays to observe interactions between NbNBR1 and βC1 at different time points. As shown at S4B Fig, the cytoplasmic granules of NbNBR1-βC1 complex became bigger and more stable at 60 hpi compared to those at 48 hpi and at 36 hpi. Western blot verified that accumulation level of NbNBR1 or βC1 at 60 hpi increased as compared to that at 48 hpi or 36 hpi by using anti-HA antibodies (S4C and S4D Fig). We also used a quantification method to support our finding by size estimation of NbNBR1-βC1 complex at different time (S4E Fig). Above all, we confirmed that NbNBR1 enhanced βC1 accumulation.

**Fig 4:**
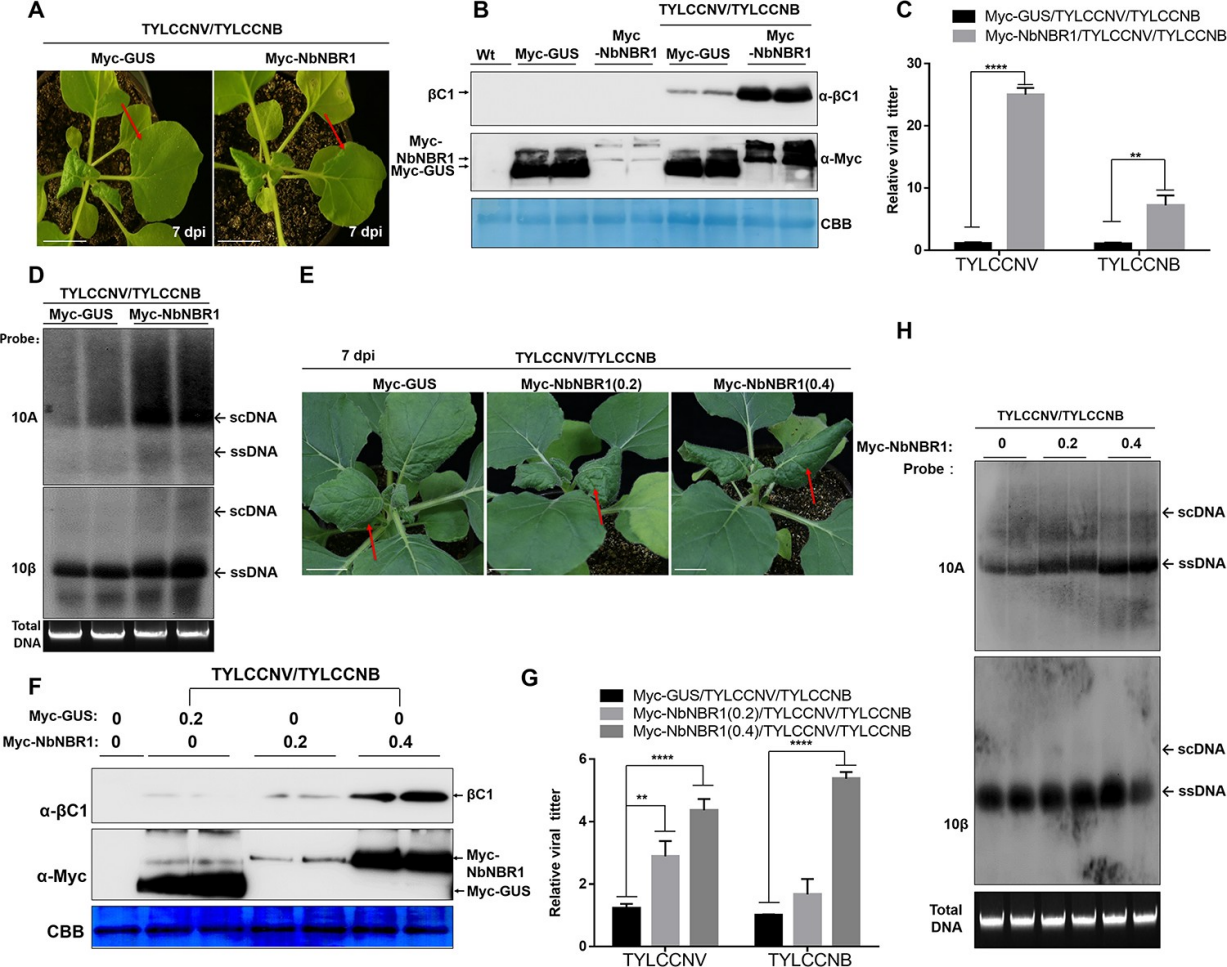
Transient over-expression of NbNBR1 promotes TYLCCNV/TYLCCNB infection. (A) Phenotypes of the *N*. *benthamiana* plants that co-expressed TYLCCNV/TYLCCNB with Myc-GUS or Myc-NbNBR1 at 7 dpi, and red arrows indicate the leaves showing TYLCCNV/TYLCCNB-induced leaf curling symptoms. Bar = 2 cm. (B) Western blot assays of the infiltrated leaves that co-expressed TYLCCNV/TYLCCNB with Myc-GUS or Myc-NbNBR1 using anti-βC1 or anti-Myc antibodies at 3 dpi. (C) qPCR analyses of viral DNA accumulations from the expanding young leaves indicated in (A). 25S rRNA was used as an internal control, and values represent the mean ± SD (n = 9). Quadruple and double asterisks indicate significant statistical differences between the two treatments (****p<0.0001, **p<0.01, Student’s *t* test). (D) Southern blot assay was used to validate the viral accumulation in the systemic leaves as indicated in (A) at 14 dpi. (E) At 7 dpi, the indicated-inoculated plants were photographed, and red arrows indicate the leaves showing TYLCCNV/TYLCCNB-induced leaf curling symptoms, Bar = 2 cm. (F) Different OD_600_ (0, 0.2, 0.4) of Myc-NbNBR1 inoculated with TYLCCNV/TYLCCNB in *N*. *benthamiana* leaves to validate influence on viral protein accumulations and viral infection at 3 dpi, anti-βC1 or anti-Myc antibodies were used in Western blot assay. (G-H) qPCR assay at 7dpi (G) and Southern blot assay at 14 dpi (H) were used to analyze TYLCCNV/TYLCCNB genomic DNA accumulations from the newly emerged leaves. Data are shown as means and SD of three biological replicates, and 25S rRNA was used as an internal control. Triple asterisks and double asterisks indicate significant differences between samples (***p<0.0001, **p<0.01, Student’s *t* test). The CBB-staining of Rubisco large subunit was set as a loading control (B, F). The Gelstain-stained agarose gel was used to show equal sample loadings, viral ssDNA and scDNA are indicated with arrows (D, H).

In the case that NbNBR1 could enhance βC1 protein level, it may affect TYLCCNV/TYLCCNB infection as feedback. We inoculated *N*. *benthamiana* leaves with TYLCCNV/TYLCCNB and Myc-NbNBR1 or Myc-GUS (control) via agroinfiltration. The TYLCCNV/TYLCCNB and Myc-NbNBR1-inoculated plants at 7 dpi showed accelerated and aggravated symptoms as compared to the plants inoculated with TYLCCNV/TYLCCNB and Myc-GUS (Fig 4A). Western blot analyses of the infiltrated leaf samples at 3 dpi showed that transient overexpression of Myc-NbNBR1 increased the accumulations of the βC1 protein (Fig 4B). qPCR and Southern blot analyses of the viral accumulation from the systemic leaves at 7 dpi (Fig 4C) and at 14 dpi (Fig 4D) showed that NbNBR1 positively regulates viral DNA accumulations. Besides, different concentrations of *A*. *tumefaciens* cultures carrying Myc-NbNBR1 (OD_600_ = 0, 0.2, 0.4) were also utilized to validate its effect on TYLCCNV/TYLCNNB infection. Consistently, the increased protein levels of NbNBR1 led to accelerated viral symptom development (Fig 4E) with more βC1 accumulations at 3 dpi (Fig 4F) and higher viral genomic DNA accumulations in systemic leaves (Fig 4G and 4H).

### Transgenic overexpression of NbNBR1 facilitates TYLCCNV/TYLCCNB infection

**Fig 5:**
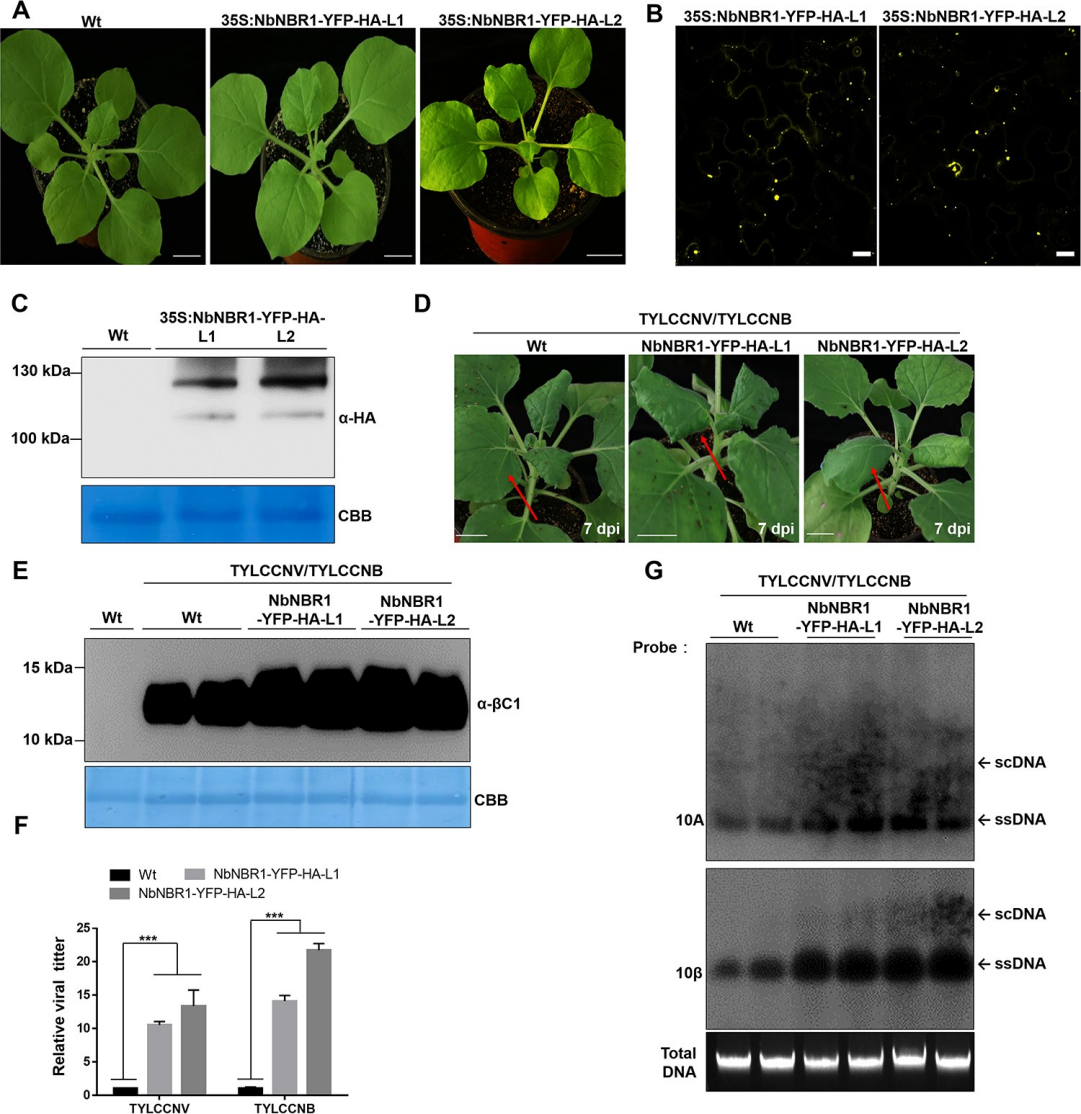
Transgenic overexpression of NbNBR1 in *N*. *benthamiana* plants enhances TYLCCNV/TYLCCNB infection. (A) Growth phenotypes of the Wt, NbNBR1-YFP-HA-L1 and NbNBR1-YFP-HA-L2 transgenic *N*. *benthamiana* plants. T1 lines were used. Bar = 2 cm. (B, C) Detection of NbNBR1-YFP-HA expression in the Wt and transgenic lines through confocal microscopy (B) or Western blot assay using anti-HA antibodies (C). Bar = 10 μm (B). (D) At 7 dpi, the inoculated plants were photographed. Bar = 2 cm. (E) Western blot analyses of βC1 accumulation in the TYLCCNV/TYLCCNB-inoculated leaves at 3 dpi, and the CBB-stained Rubisco large subunit gel was used to show sample equal loadings. (F) qPCR analyses of viral DNA accumulations in the expanding young leaves at 7 dpi. 25S rRNA was used as an internal control, and the data presented are the means ± SD (n = 9). Triple asterisks indicate significant statistical differences between treatments (***p<0.001, Student’s *t* test). (G) Southern blot analyses of the viral accumulation in the systemic leaves at 14 dpi. The Gelstain-stained agarose gel was used to show equal sample loadings, and viral ssDNAs and scDNA are indicated with arrows.

Furthermore, we generated two NbNBR1-YFP-HA transgenic *N*. *benthamiana* T1 lines (NbNBR1-YFP-HA-L1 and NbNBR1-YFP-HA-L2) and inoculated them with TYLCCNV/TYLCCNB through agroinfiltration. Growth phenotypes of the two transgenic lines were similar to that shown by the Wt plants (Fig 5A). Yellow fluorescence could be observed under a confocal microscope from leaf samples of these two transgenic lines (Fig 5B). Western blot assay using anti-HA antibodies further confirmed the expression of NbNBR1-YFP-HA in these two transgenic lines (Fig 5C). After inoculation with TYLCCNV/TYLCCNB, much severe disease symptoms appeared on *NbNBR1*-transgene *N*. *benthamiana* plants at 7 dpi compared to those in the Wt plant (Fig 5D). At the same time, the NbNBR1-YFP-HA transgenic plants accumulated more βC1 protein levels than the Wt plants in the infiltrated leaves at 3 dpi (Fig 5E). qPCR analyses at 7 dpi and Southern blot analyses at 14 dpi of viral DNA accumulations in the systemic leaves of plants indicated in Fig 5D, showed higher viral DNAs accumulated in the NbNBR1-YFP-HA-overexpressing *N*. *benthamiana* plants than that in the Wt plants (Fig 5F and 5G). Meanwhile, we inoculated these two NbNBR1-YFP-HA transgenic *N*. *benthamiana* lines with PVX or PVX-βC1. Overexpression of NbNBR1 did not affect PVX infection (S5A and S5B Fig). However, at 5 dpi, NbNBR1-overexpressing lines inoculated with PVX-βC1 showed much severe disease symptoms together with increased βC1 protein level as compared to the Wt plants (S5C and S5D Fig). Additionally, we used qRT-PCR to check mRNA level of βC1 on TYLCCNV/TYLCCNB infected NbNBR1-YFP-HA transgenic plants at 2 dpi, no marked change was found, confirming that NbNBR1 affected the level of βC1 protein rather than mRNA (S5E Fig). Above data suggest that NbNBR1 is a susceptible factor for TYLCCNV/TYLCCNB infection.

### βC1 escapes the NbRFP1-mediated degradation in the presence of NbNBR1

**Fig 6:**
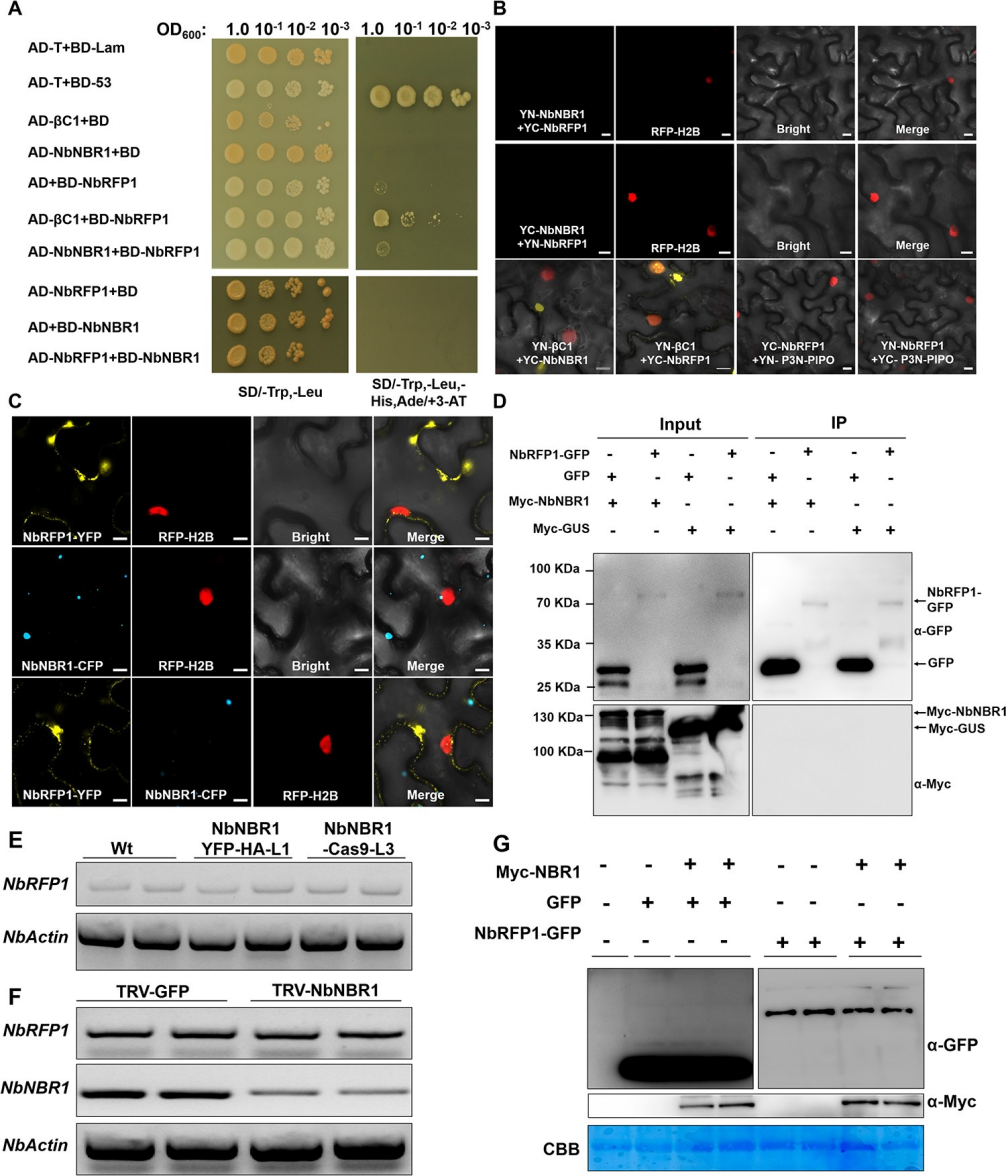
No direct interaction between NbNBR1 and NbRFP1, and NbNBR1 does not affect *NbRFP1* mRNA and protein expressions. (A) Y2HGold cell cultures transformed with the indicated constructs were first serially diluted (1.0 to 10^−3^) and then grown on the SD-Trp-Leu-His-Ade medium supplemented with 10 mM 3-aminotriazole (3-AT). (B) *A*. *tumefaciens* cultures carrying the indicated constructs were individually infiltrated into the leaves of the RFP-H2B transgenic *N*. *benthamiana* plants. The infiltrated leaf cells were examined and imaged under a confocal microscope at 48 hpi. Bar = 10 μm. (C) *A*. *tumefaciens* cultures carrying NbRFP1-YFP, NbNBR1-CFP or NbRFP1-YFP+NbNBR1-CFP were infiltrated into the RFP-H2B transgenic *N*. *benthamiana* leaves, respectively, followed by confocal microscopy. Bar = 10 μm. (D) Co-IP assay was utilized to determine the interaction between Myc-NbNBR1 and NbRFP1-GFP. *A*. *tumefaciens* cultures carrying the indicated constructs were infiltrated into *N*. *benthamiana* leaves, respectively. Samples made before (Input) and after (IP) immunoprecipitation were analyzed through Western blot assays using anti-GFP or anti-Myc antibodies. (E, F) End point qPCR was conducted to determine the expressions of *NbNBR1* and *NbRFP1* in the NbNBR1-YFP-HA transgenic, NbNBR1-Cas9 mutant, or *NbNBR1*-silenced plants. (G) Western blot analyses were used to determine the expression levels of Myc-NbNBR1 and NbRFP1-GFP using anti-GFP or anti-Myc antibodies. The CBB-stained Rubisco large subunit gel was used to show equal sample loadings. Each experiment was performed three times.

Our previous study revealed NtRFP1 could bind and ubiquitinate βC1 to mediate their degradation [31]. Because NBR1 as a selective autophagic adaptor recognizes ubiquitinated protein complexes [5], we hypothesized that the ubiquitinated NbRFP1 might be a substrate of NbNBR1. To test this hypothesis, we investigated the interaction between NbNBR1 and NbRFP1 (a homolog of NtRFP1 in *N*. *benthamiana*) using Y2H, BiFC, and Co-IP assays. The result of Y2H assay showed that the yeast cells co-transformed with AD-NbRFP1+BD-NbNBR1 or AD-NbNBR1+BD-NbRFP1 failed to grow on the selective medium supplemented with 10 mM 3-amino-1,2,4-triazole (3-AT), while yeast cells co-transformed with AD-βC1+BD-NbRFP1 did (Fig 6A). Similarly, no interaction was found between NbNBR1 and NbRFP1 in BiFC, and Co-IP assays, and no co-localization was observed between NbRFP1-YFP and NbNBR1-CFP in plant cell (Fig 6B–6D), indicating that NbNBR1 and NbRFP1 did not interact *in vivo*. In order to know if NbNBR1 affects the expression level of NbRFP1, we analyzed *NbRFP1* mRNA expression level in NbNBR1-YFP-HA transgenic lines, NbNBR1-Cas9 transgenic lines and TRV-NbNBR1 inoculated lines. No change of *NbRFP1* mRNA expression level in the above plants compared to control plants was found (Fig 6E and 6F). Besides, co-expression of Myc-NbNBR1 and NbRFP1-GFP in *N*. *benthamiana* was used to investigate NbRFP1 protein level by anti-GFP antibodies. As shown in Fig 6G, no specific variation of NbRFP1 was found either when co-expression with Myc-NbNBR1 or not (Fig 6G). These results indicate that NbNBR1-mediated accumulation of βC1 is not because of altering the expression level of NbRFP1.

**Fig 7:**
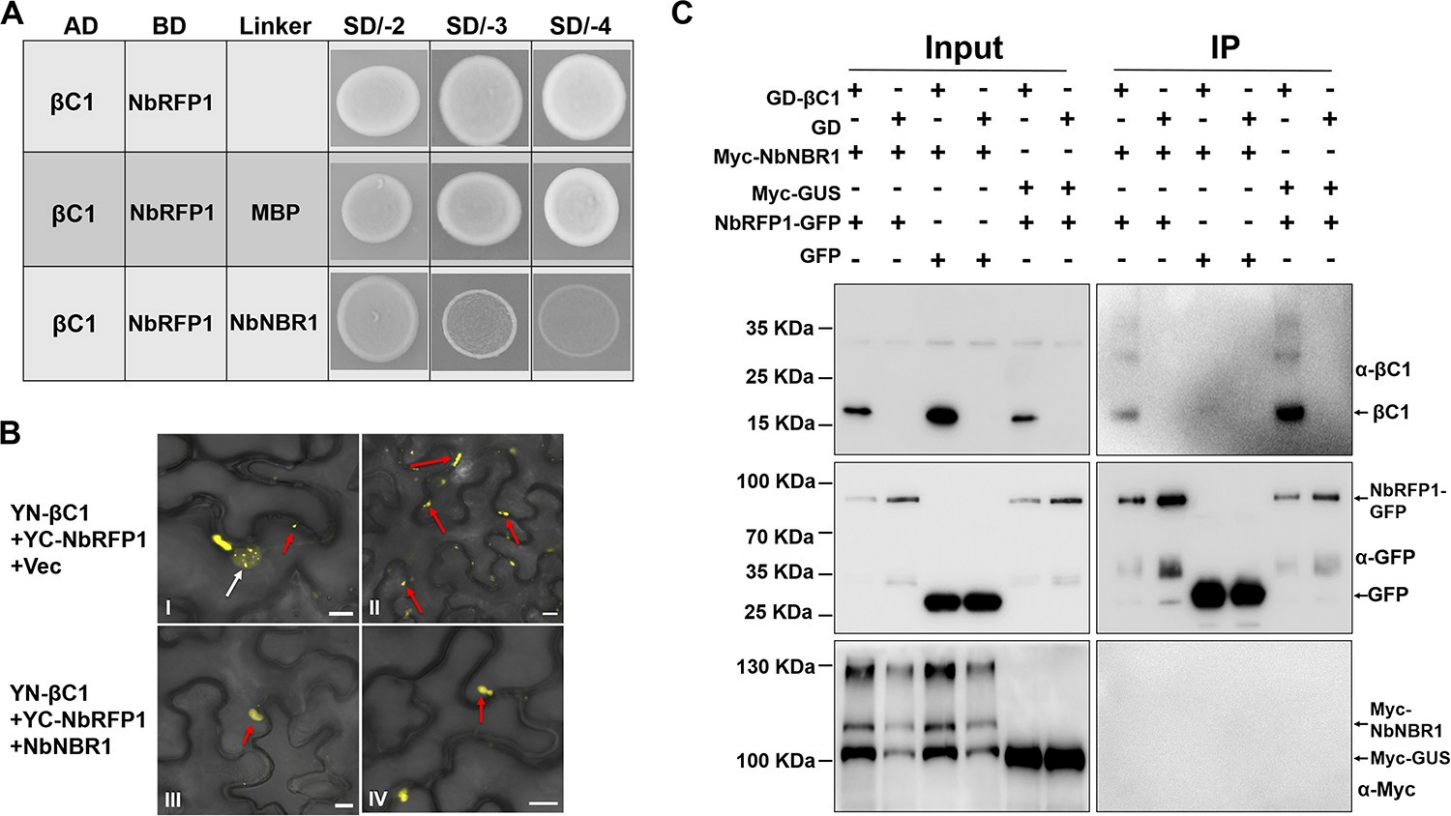
NbNBR1 prevents βC1 degradation by perturbing the interaction of NbRFP1 and βC1. (A) Y3H assay was used to determine the interaction among βC1, NbRFP1, and NbNBR1. Yeast cell culture co-expressing AD-βC1+Bridge-NbRFP1-MBP was used as a control. Different yeast cell cultures transformed with the indicated plasmids were grown on the SD/-Leu-Trp (SD/-2), SD/-Leu-Met-Trp (SD/-3), and SD/-His-Leu-Met-Trp (SD/-4) media, respectively. (B) BiFC assay was performed to determine the subcellular localization patterns of YN-βC1 and YC-NbRFP1 without NbNBR1 (panel I and II) or with NbNBR1 (panel III and IV) in *N*. *benthamiana* leaf cells. Red arrows indicate granules in the cytoplasm while the white arrow indicates the nucleus. Bar = 10 μm. (C) Immunoprecipitation assays were performed to determine the accumulation levels of GD-βC1, NbRFP1-GFP, and Myc-NbNBR1. Protein samples made from the infiltrated leaves with expressing the indicated plasmids before (Input) and after (IP) immunoprecipitation were analyzed through Western blot assays using anti-βC1, anti-GFP or anti-Myc antibodies.

Based on these results, we thus speculated that NbNBR1 can compete with NbRFP1 for βC1. To test this, we firstly investigated the interaction among the three proteins through Y3H assay. We found that NbRFP1 interacted with βC1, and this interaction was not affected in the presence of MBP (Fig 7A). In contrast, the presence of NbNBR1-MBP inhibited the interaction between βC1 and NbRFP1. Next, we conducted confocal imaging to check whether NbNBR1 could change the interaction site and affect the interaction ability of NbRFP1 and βC1 in *N*. *benthamiana* cells. As shown in Panel I of Fig 7B, yellow fluorescence from infiltrated leaves with pairwise expression of YN-βC1 and YC-NbRFP1 was present in the nucleus and in the cytoplasm. While we noticed that the yellow fluorescence signals generated from the interaction complex of YN-βC1 and YC-NbRFP1 only localized in the cytoplasm when in the presence of NbNBR1 (Fig 7B, compare Panel I with Panel III). Meanwhile, less interaction granules of YN-βC1 and YC-NbRFP1 in the cytoplasm were found in the expression of NbNBR1 (Fig 7B, compare Panel II with Panel IV). To further validate the relationship of NbNBR1, NbRFP1, and βC1 in plant, we co-expressed GD-βC1+Myc-NbNBR1+NbRFP1-GFP (Fig 7C, 1st lane), GD+Myc-NbNBR1+NbRFP1-GFP (Fig 7C, 2nd lane), GD-βC1+Myc-NbNBR1+GFP (Fig 7C, 3rd lane), GD+Myc-NbNBR1+GFP (Fig 7C, 4th lane), GD-βC1+Myc-GUS+NbRFP1-GFP (Fig 7C, 5th lane), GD+Myc-GUS+NbRFP1-GFP (Fig 7C, 6th lane), and the corresponding protein accumulations were analyzed using anti-βC1, anti-Myc and anti-GFP antibodies. The result showed that much more βC1 accumulated in the extracts containing GD-βC1, Myc-NbNBR1, and NbRFP1-GFP than the extracts containing GD-βC1, Myc-NbNBR1 and GFP (Fig 7C, compare the 1st lane with the 5th lane in input Western blot using anti-βC1 antibodies), suggesting that the presence of NbNBR1 can protect βC1 from the NbRFP1-mediated degradation. In addition, less GD-βC1 was immunoprecipitated using GFP-Trap_MA magnetic agarose beads from the extracts containing GD-βC1+Myc-NbNBR1+NbRFP1-GFP (Fig 7C, 1st lane) than the extracts containing GD-βC1+Myc-GUS+NbRFP1-GFP (Fig 7C, 5th lane), supporting that NbNBR1 could interfere the interaction between NbRFP1 and βC1. Furthermore, we analyzed the ubiquitination level of the total protein after TYLCCNV/TYLCCNB infection in both NbNBR1-overexpression transgenic lines (NbNBR1-YFP-HA-L1/L2) and NbNBR1-Cas9 lines (NbNBR1-Cas9-L3/L4). As expected, overexpression of NbNBR1 reduced the ubiquitination level of total protein, while vice versa (S6A and S6B Fig).

### A single amino acid substitution in βC1 (βC1^K4A^) abolishes its interaction with NbNBR1 and attenuates TYLCCNV/TYLCCNB infection

**Fig 8:**
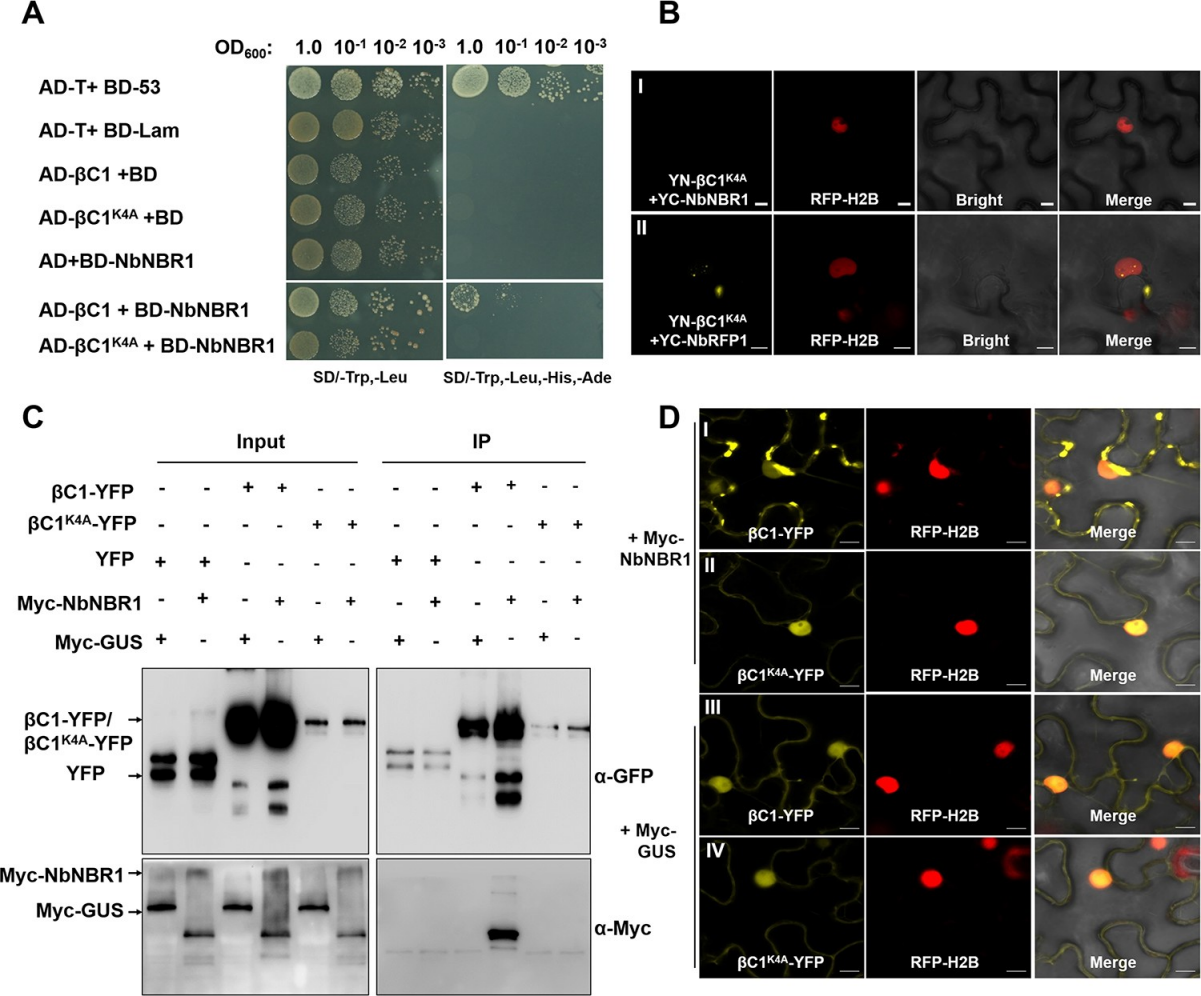
A single amino acid substitution in βC1 (βC1^K4A^) abolishes the interaction between βC1 and NbNBR1. (A) Yeast cell cultures transformed with the indicated plasmids were 10-fold diluted and then grown on the SD/-Trp-Leu or SD/-Trp-Leu-His-Ade medium. Yeast cell culture co-transformed with AD-βC1+BD-NbNBR1 was used as the positive control. (B) YN-βC1^K4A^+YC-NbNBR1, and YN-βC1^K4A^+YC-NbRFP1 were co-expressed, respectively, in RFP-H2B transgenic *N*. *benthamiana* leaves followed by confocal microscopy at 48 hpi. Bar = 10 μm. (C) Co-IP assay was performed to validate the interaction between Myc-NbNBR1 and βC1-YFP, and Myc-NbNBR1 and βC1^K4A^-YFP. Input and IP represent the samples made before and after immunoprecipitation, respectively. Western blot assays were performed using anti-GFP and anti-Myc antibodies. (D) βC1-YFP and βC1^K4A^-YFP were, respectively, co-expressed with Myc-NbNBR1 or Myc-GUS in RFP-H2B transgenic *N*. *benthamiana* leaves followed by confocal microscopy at 48 hpi. Bar = 10 μm. The protein expression of the integrity of fused proteins was confirmed in S7C Fig.

To further investigate the interaction among NbNBR1, βC1, and NbRFP1, we mutated βC1 through truncated mutation and site-directed mutagenesis. Firstly, we divided βC1 into 12 fragments, and found the first fragment (1–10 aa) of βC1 is required for its interaction with NbNBR1 (S7A and S7B Fig). Then we made a series of amino acid site mutations, and found that βC1 with the fourth lysine mutation no longer interacted with NbNBR1 in yeast cells (Fig 8A). Under the confocal microscope, no YFP fluorescence was observed in the RFP-H2B transgenic *N*. *benthamiana* leaf cells when co-expressing YN-βC1^K4A^ and YC-NbNBR1 (Fig 8B). Co-IP assay also showed that βC1^K4A^ and Myc-NbNBR1 were not immunoprecipitated together any more (Fig 8C). However, this mutation did not interfere with the interaction between βC1and NbRFP1 (Fig 8B). It is noteworthy that co-expression of Myc-NbNBR1+βC1-YFP in cells increased the intensity of βC1-YFP fluorescence, while the increased yellow fluorescence was not seen in the cells that co-expression of Myc-NbNBR1+βC1^K4A^-YFP (Fig 8D), suggesting that the interaction between NbNBR1 and βC1 stabilized βC1.

**Fig 9:**
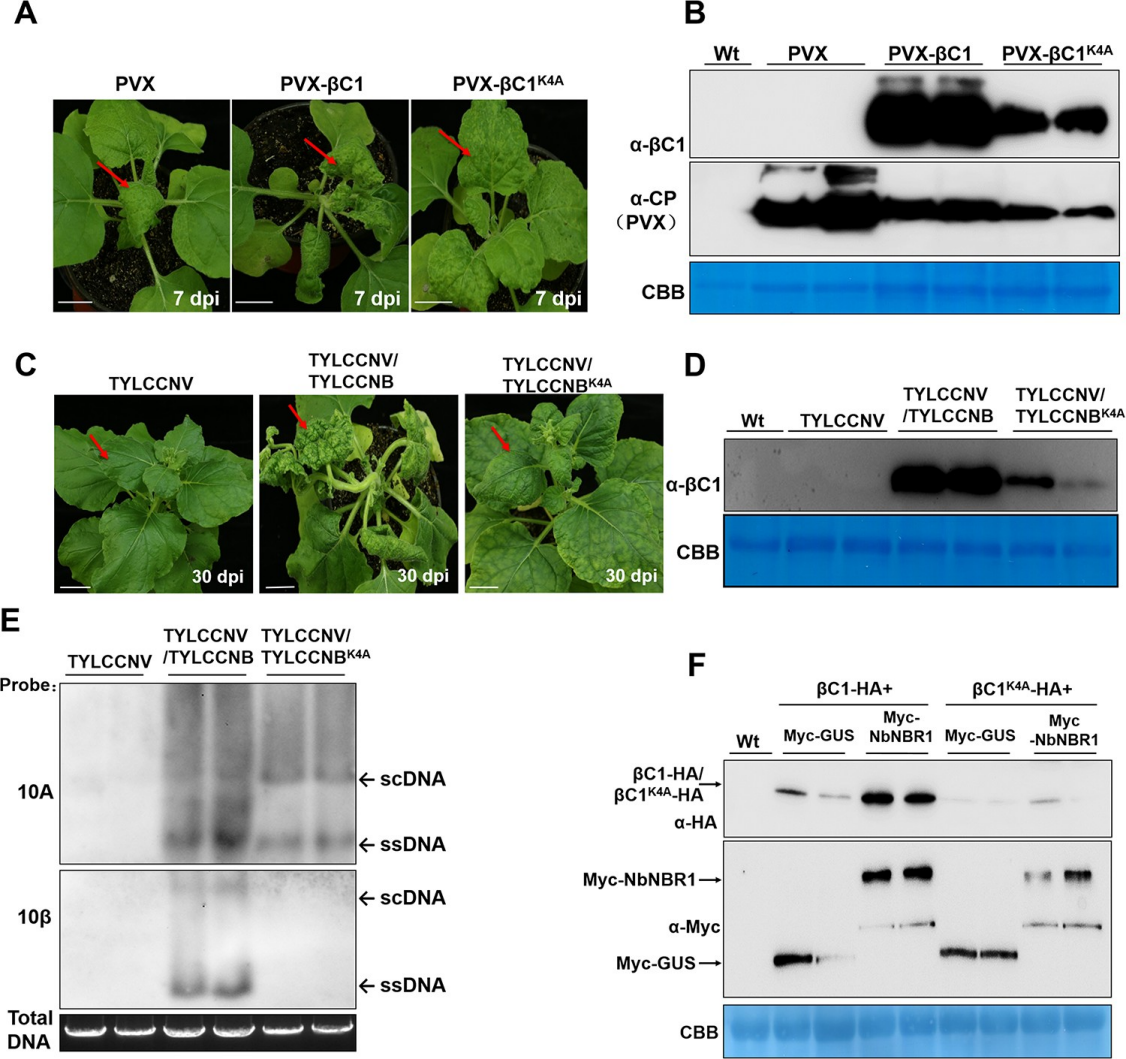
Disruption of βC1 and NbNBR1 interaction attenuates TYLCCNV/TYLCCNB infection. (A) Disease symptoms shown by the PVX-, PVX-βC1-, and PVX-βC1^K4A^-infected *N*. *benthamiana* plants at 7 dpi, respectively. Red arrows indicate the leaves showing mosaic and leaf curling symptoms. Bar = 2 cm. (B) Western blot analyses of βC1 and PVX CP accumulations in the plants shown in (A) using anti-βC1 or anti-PVX CP antibodies. The CBB-stained Rubisco large subunit gel was used to show equal sample loadings. (C) Disease symptoms of TYLCCNV-, TYLCCNV/TYLCCNB-, and TYLCCNV/TYLCCNB^K4A^-infected *N*. *benthamiana* plants were photographed at 30 dpi, and red arrowheads indicate the leaves showing mottling and leaf distortion symptoms, Bar = 2 cm. (D) Western blot analyses of βC1 accumulation in the TYLCCNV-, TYLCCNV/TYLCCNB-, and TYLCCNV/TYLCCNB^K4A^-inoculated leaves at 3 dpi, and the CBB-stained Rubisco large subunit gel was used to show equal sample loadings. (E) Southern blot analyses of viral DNA accumulations in the systemic leaves of the assayed plants shown in (C) at 30 dpi. The Gelstain-stained agarose gel was used to show equal sample loadings, viral ssDNA and scDNA are indicated with arrows. (F) Western blot analyses of Myc- or HA-tagged proteins as indicated in the samples prepared from the inoculated *N*. *benthamiana* leaves at 48 hpi. The blots were probed with anti-Myc or anti-HA antibodies. The CBB-stained Rubisco large subunit gel was used to show equal sample loadings.

To investigate the role of βC1 and NbNBR1 interaction in βC1-induced symptoms, PVX-βC1 and PVX-βC1^K4A^ were inoculated individually to *N*. *benthamiana* plants. By 7 dpi, the PVX-βC1-inoculated plants showed strong mosaic and leaf curling symptoms (Fig 9A). Although the PVX-βC1^K4A^-inoculated plants also showed strong mosaic symptoms, they did not show strong leaf curling symptoms and Western blot result showed PVX-βC1^K4A^-inoculated plants accumulated much less βC1 as compared with the PVX-βC1-inoculated plants (Fig 9A and 9B). We then introduced the K4A mutation into the TYLCCNB (TYLCCNB^K4A^) and inoculated TYLCCNV/TYLCCNB and TYLCCNV/TYLCCNB^K4A^, respectively, to *N*. *benthamiana* plants through agroinfiltration. By 30 dpi, the TYLCCNV/TYLCCNB^K4A^-inoculated plants displayed attenuated disease symptoms (Fig 9C). Western blot result showed that the TYLCCNV/TYLCCNB^K4A^-inoculated plants also accumulated much less βC1 than the TYLCCNV/TYLCCNB-inoculated plants at 3 dpi (Fig 9D) and Southern blot assay at 30 dpi (Fig 9E) showed that less viral genomic DNAs in Fig 9C when compared to the TYLCCNV/TYLCCNB-inoculated plants. Furthermore, co-expression of Myc-NbNBR1 and βC1^K4A^-HA in *N*. *benthamiana* leaves resulted in a much lower level of βC1^K4A^ than that in the leaves co-expression of Myc-NbNBR1 and βC1-HA (Fig 9F). Taken together, these results indicate that the interaction between βC1 and NbNBR1 is required for the βC1 protein accumulation and βC1-dependent viral pathogenicity.

## Discussion

Autophagy was initially shown to regulate plant immunity to DNA and RNA viruses, including CaMV, CLCuMuV, TYLCCNV, TMV, TuMV, cucumber mosaic virus, rice stripe virus and pepper mild mottle virus [15–18,33–36]. To counteract autophagy-mediated host resistance, many RNA viruses have evolved specific abilities to manipulate host autophagy machinery directly or indirectly to benefit their infections [18, 19, 21, 37–38]. In 2019, Huang and colleagues reported that the *Arabidopsis* chloroplast-derived vesicles contain ATG8f, an autophagy cargo receptor/adaptor protein, and functioned as shelters for BaMV replication [20]. Rice gall dwarf virus has been shown to induce virion containing autophagosomes that can facilitate virus transmission through insect vectors [39].

NBR1 was initially reported as a selective autophagy receptor that has a typical conserved LC3 (microtubule-associated protein 1 light chain 3)-interacting region (LIR) and an ubiquitin (Ub)-binding domain (UBA) and can be recruited to ubiquitinate its targets. *Arabidopsis* AtNBR1 is involved in autophagy-associated degradation of vacuoles and contains two UBA domains [40]. *N*. *tabacum* NtJoka2 is a homolog of AtNBR1, and acts as a selective receptor to inhibit the colonization of potato blight pathogen, *Phytophthora infestans* [41–42]. Here we showed a geminivirus satellite-encoded protein could upregulate *NbNBR1* expression when transgenic overexpression and transient overexpression of βC1 in *N*. *benthamiana* leaves (S8 Fig), and interact with NbNBR1 to form cytoplasmic granules. These cytoplasmic granules can prevent βC1 from being degraded by the NbRFP1-mediated UPS-dependent degradation system to facilitate geminivirus infection, which demonstrated a distinct function of NBR1. NBR1 as an autophagy adaptor can participate in the autophagic degradation of its substrates. However, co-expression of NbNBR1 and βC1 in our study failed to degrade βC1 but increased its accumulation (Fig 3). Consistent with this result, transient or stable overexpression of NbNBR1 in *N*. *benthamiana* enhanced βC1 virulence, leading to stronger viral symptoms (Figs 4 and 5), and vice versa (Fig 2). In addition, overexpression of NbNBR1 in *N*. *benthamiana* also promoted the infection of another monopartite geminivirus: tomato yellow leaf curl virus Beijing isolate (TYLCV-BJ) (S9 and S10 Figs), and its V2 protein interacted with NbNBR1 and formed cytoplasmic granules as well (S11 Fig). These findings suggest that the NbNBR1-mediated pro-viral role may be a general phenomenon during geminivirus infections.

NtRFP1 can be ubiquitinated as a substrate of the UPS-dependent degradation machinery and mediate the degradation of βC1 [31], and it may be recognized by NBR1. However, we did not observe a direct interaction between NbNBR1 and NbRFP1 through Y2H, BiFC, co-localization and Co-IP assays (Fig 6). Y3H assay showed that NbNBR1 competed with NbRFP1 for βC1, which disrupted the interaction between NbRFP1 and βC1 (Fig 7A), indicating a different fate of βC1 when it is hijacked by RFP1 or NBR1 in plant. A mutant βC1 with a single amino acid substitution (βC1^K4A^) which failed to interact with NbNBR1 but it could still interact with NbRFP1, was found in this study (Fig 8). We found less βC1 accumulations and milder βC1-induced viral symptoms in PVX-βC1^K4A^ mutant in contrast to wild type PVX-βC1 -infected plants (Fig 9A and 9B). Similarly, the TYLCCNV/TYLCCNB^K4A^ also displayed much weaker virulence and accumulated less viral genomic DNA compared to wild type TYLCCNV/TYLCCNB in infected plants (Fig 9C–9E), supporting the interaction between βC1 and NbNBR1 contributes to the stability and virulence of βC1.

**Fig 10:**
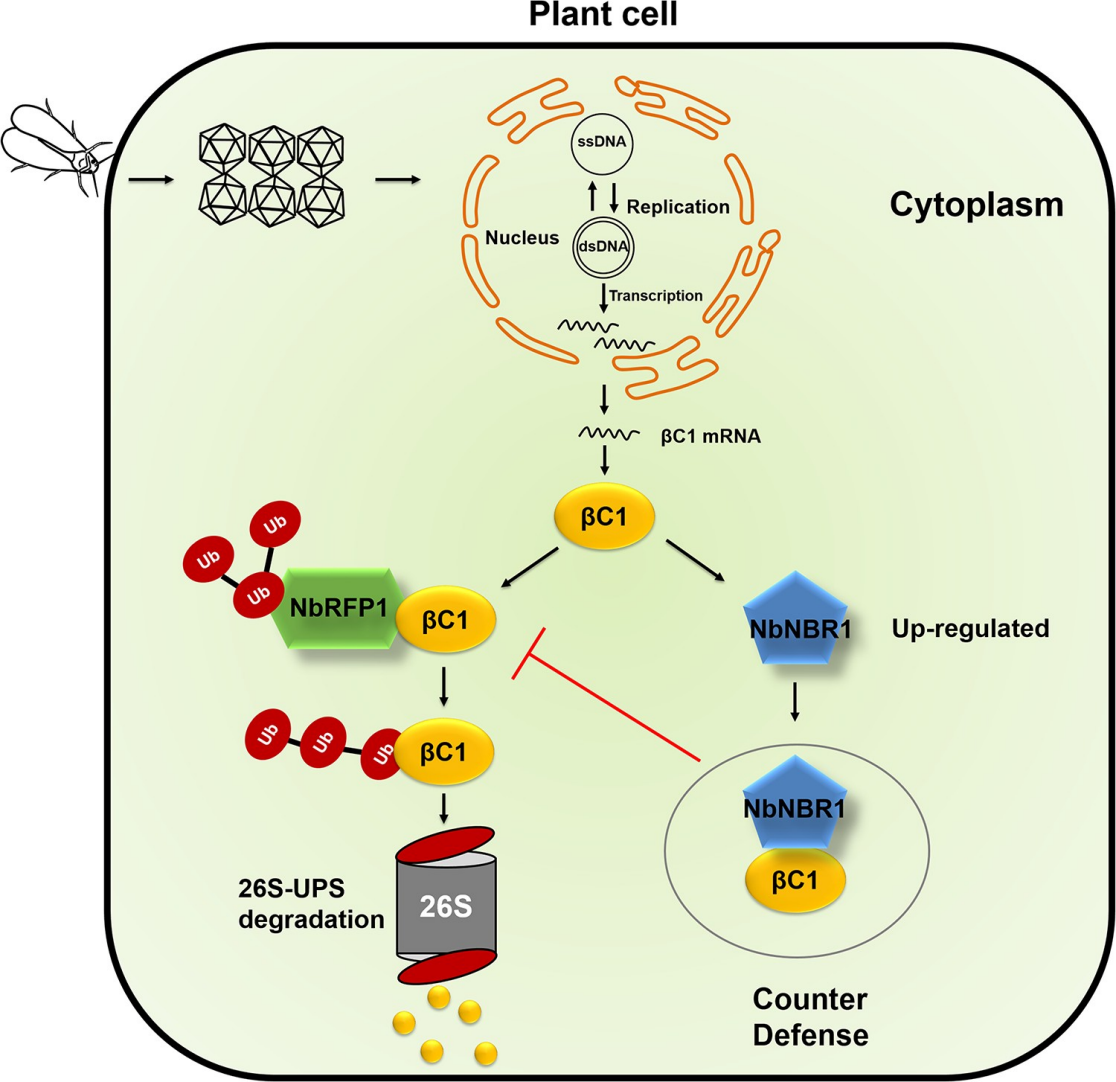
A working model for the function of NbNBR1 in geminivirus infection in plant. Geminivirus-encoded βC1 is a pathogenicity determinant and a target of plant defense machinery. Geminivirus βC1 could be degraded by the NbRFP1-mediated ubiquitin-26S proteasome system (UPS) [31]. To counteract host defense, βC1 induces the expression of *NbNBR1* and interacts with it in the cytoplasm to form granules. These cytoplasmic granules can prevent βC1 from being degraded by the NbRFP1-mediated UPS-dependent degradation, leading to an increased βC1 accumulation and much severe disease symptoms.

Based on the above results, we proposed a working model for the interactions among NbNBR1, NbRFP1, and βC1 during geminivirus infection in plant (Fig 10). Geminiviruses are delivered into plant cells by insect vectors. Inside the infected cells, viral proteins are translated and become the targets of host surveillance. The NbRFP1-mediated UPS-dependent degradation system recognizes, interacts with, and then degrades the viral betasatellite-encoded βC1, leading to an inhibition of virus accumulation and disease symptom formation [26]. To counteract host defense, βC1 interacts with NbNBR1 to form cytoplasmic granules to prevent βC1 degradation by NbRFP1. The finding of NbNBR1, NbRFP1, and βC1 three-way-interactions expands our knowledge on the arm races between plant and geminiviruses. In addition, our finding also suggests that *NbNBR1* is a susceptible host gene to geminivirus invasion, which might be applied into molecular breeding in other crops for restricting viral infection.

## Materials and methods

### Plant materials and growth conditions

Wild type (Wt), RFP-H2B transgenic, βC1 transgenic, NbNBR1-YFP-HA transgenic, and NbNBR1-Cas9 mutant *N*. *benthamiana* seedlings were grown in pots inside an insect-free greenhouse maintained at 22°C/18°C (day/night), 16 h light/8 h dark photoperiod, and 60% relative humidity. These plants were used for assays after they grew to 4–5 leaves.

### Plasmid construction

*NbRFP1* sequence was amplified and cloned behind the 35S promoter in pCHF3 predigested with *Sac*I and *BamH*I restriction enzyme to produce pCHF3-NbRFP1-GFP (referred to as NbRFP1-GFP thereafter). Plasmid pBA-Flag-Myc4-NbNBR1 (referred to as Myc-NbNBR1 thereafter), pBA-Flag-Myc4-GUS (referred to as Myc-GUS thereafter), pNbRFP1-YFP (NbRFP1-YFP), pYN-NbRFP1 (YN-NbRFP1), and pYC-NbRFP1 (YC-NbRFP1) were constructed, respectively, using the Gateway system as instructed (Invitrogen, Carlsbad, CA, USA). Plasmid pCambia1300-βC1-GFP (βC1-GFP), pGD-βC1 (GD-βC1), and pPVX-βC1 (PVX-βC1) were constructed as described previously [43]. Plasmid pYN-NbNBR1 (YN-NbNBR1), pYC-NbNBR1 (YC-NbNBR1), and pNbNBR1-CFP (NbNBR1-CFP) were constructed, respectively, using PCR as described [21]. Primers used in this study are listed in S1 Table. The site-directed mutant plasmid pPVX-βC1^K4A^ (PVX-βC1^K4A^) and pGD-βC1^K4^-HA (βC1^K4^-HA) were generated using the Mut Express II Fast Mutagenesis Kit V2 as instructed (Vazyme, Nanjing, CHN). Plasmid pTYLCCNB^K4A^ (pTYLCCNB^K4A^) was made using a published protocol [44]. Plasmid pBridge-NbRFP1 (Bridge-NbRFP1), pBridge-NbRFP1-NbNBR1 (Bridge-NbRFP1-NbNBR1), and pBridge-NbRFP1-MBP (Bridge-NbRFP1-MBP) were constructed as described previously [45]. NbNBR1-Cas9 construct was obtained as followings: The ideal spacer was reference to the CRISPR-P online website (http://cbi.hzau.edu.cn/cgi-bin/CRISPR). SgRNA sequence is ACACAGCACTGAATGATGCA. NbNBR1-spacer1-F/R primes were designed according to the sequence of sgRNA (S1 Table). After annealing, the double fragments were directly connected with BKG01 vector to obtain BKG01-NbNBR1, which was confirmed by DNA sequencing. *Agrobacterium tumefaciens* strain EHA105 cells were transformed with individual expression vectors, while *A*. *tumefaciens* strain GV3101 cells were transformed with individual PVX-based vectors.

### Yeast two-hybrid (Y2H), bimolecular fluorescence complementation (BiFC), and subcellular localization assays

Full-length coding sequences of TYLCCNB βC1, NbNBR1 and NbRFP1 were amplified by PCR and cloned individually into pGADT7 or pGBKT7 vector to produce pGAD-βC1 (AD-βC1), pGAD-NbNBR1 (AD-NbNBR1), pGBD-NbNBR1 (BD-NbNBR1), and pGBD-NbRFP1 (BD-NbRFP1), respectively. For Y2H assays, different combinations of plasmids were transformed into Y2HGold strain cells. The transformed cell cultures were grown individually on the selective medium (SD) with -Leu-Trp for 96 h at 30°C, and then on selective medium with SD/-Leu-Trp-His-Ade to determine the interactions between NbNBR1 and βC1 or βC1^K4A^. The selective medium with SD/-Leu-Trp-His-Ade and 10 mM 3-aminotriazole (3-AT) was used to determine the interactions between NbRFP1 and NbNBR1. In this study, cell cultures co-transformed with AD-T7-T and BD-T7-53 (referred to as AD-T+BD-53 thereafter) or AD-T7-T and BD-T7-Lam (AD-T+BD-Lam) were used as the positive and negative controls, respectively. For Y3H assays different combinations of plasmids were transformed into AH109 strain cells using the Yeast Transformation II kit as instructed (ZYMO, USA). The transformed cell cultures were grown individually on the selective medium (SD) with -Leu-Trp for 96 h at 30°C, and then on selective medium with SD/-Leu-Met-Trp (SD/-3), and SD/-His-Leu-Met-Trp (SD/-4) media, respectively.

For BiFC and subcellular localization assays, the excitation wavelength of CFP was set at 458 nm and the emission was captured at 470–500 nm. The excitation wavelength of YFP was set at 514 nm and the emission was captured at 565–585 nm. The excitation wavelength of GFP was set at 488 nm and the emission was captured at 510–550 nm, while the excitation wavelength of RFP was set at 543 nm and the emission was captured at 590–630 nm as described [7]. Epidermal cells of the assayed leaves were harvested at 48 and 72 hours post infiltration (hpi) and examined under a confocal laser scanning microscopy 980 (Zeiss, GRE).

### Virus inoculation and agroinfiltration

Virus infectious clones: pBinPLUS-Y10-1.7A (referred to as TYLCCNV) [25], pBinPLUS-Y10-1.7A+Y10β (TYLCCNV/TYLCCNB) [44], and pCambia2300-1.4A (TYLCV-BJ) (Genbank accession number MN432609), were transformed individually into *A*. *tumefaciens* cells. The transformants were cultured and then diluted to OD_600_ = 0.4. Equal volumes of the diluted cultures were mixed, as indicated in figure legends, before infiltration into *N*. *benthamiana* leaves. The infiltrated plants were photographed with a Canon 530D digital camera at various days post inoculation (dpi).

For virus-induced gene silencing (VIGS) assay, we used TRV-based VIGS vectors [pTRV1 and pTRV2-NbNBR1 (TRV1+TRV-NbNBR1) or pTRV1 and pTRV2-GFP (TRV1+TRV-GFP, control vector)]. *A*. *tumefaciens* culture carrying TRV1 was diluted in the infiltration buffer (10 mM MgCl_2_, 10 mM MES, pH5.6, and 100 μM acetosyringone) till OD_600_ = 0.5, while *A*. *tumefaciens* cultures carrying TRV2-GFP or TRV2-NbNBR1 were diluted to OD_600_ = 0.6. Equal volumes of *A*. *tumefaciens* cultures carrying TRV1 or TRV-NbNBR1, or TRV1 or TRV-GFP were mixed prior to infiltration.

### DNA extraction and Southern blot analyses

Total DNA was extracted from leaf samples using the CTAB method. After denaturation and neutralization, total DNA was separated in agarose gels through electrophoresis and then transferred to Hybond N^+^ nylon membranes (GE Healthcare, Pittsburgh, PA, USA). The membranes were hybridized at 55 ^o^C with digoxin-labeled probes prepared using the DIG High Prime DNA Labeling and Detection Starter Kit (Roche, Mannheim, GRE). Agarose gels stained with GelStain (TransGen Biotech, Beijing, CHN) were used to show equal sample loadings.

### Quantitative polymerase chain reaction (qPCR) and quantitative reverse transcription PCR (qRT-PCR)

Total genomic DNA (gDNA) and total RNA (T. RNA) were extracted, respectively, from the infiltrated and expanding young leaves using the CTAB method or Trizol reagent (Invitrogen, Carlsbad, CA, USA). For qPCR, 100 ng gDNA was used in each 10 μL reaction. For qRT-PCR, 1000 ng T. RNA was used in a 10 μL RT reaction made with a reverse transcription kit (TAKARA, JPN) followed by qPCR using the universal SYBR Green Master kit (TAKARA, JPN). The expression levels of *25S rRNA* and *F-box* mRNA were used as the internal controls for qPCR and qRT-PCR assays, respectively. At the end of each run, melting curve was used to assess the specificity of amplification product (60 to 95°C with heating rate at 0.5°C for 10 s and measure fluorescence continuously).

### Transient gene expression and Western blot assays

Transient gene expression assays were performed in *N*. *benthamiana* leaves through agroinfiltration as described above. Total protein was extracted from individual leaf samples homogenized in the extraction buffer and separated in gels through electrophoresis. After transferring to blotting membranes, protein bands were detected using anti-GFP (Roche, Mannheim, GRE) or anti-Myc (Genscript, Piscataway, NJ, USA) polyclonal antibodies, or anti-HA (Roche, Mannheim, GRE) or anti-βC1 (made in our laboratory) monoclonal antibodies.

## Supporting information

S1 Fig Co-localization analyses of the βC1-NbNBR1 interaction complex with the markers of sub-cellular compartments, and Western blot confirmation of expression of the fused proteins in Fig 1D. (A) YN-βC1 and YC-NbNBR1 were co-expressed by agrobacteria-mediated infiltration with some reported markers of sub-cellular compartments [Endosome: Sec2 as a marker, chloroplast, peroxisome: SK4 as a marker, Golgi, and endoplamic reticulum (ER)] in *N*. *benthamiana* leaves at 48 hpi. Bar = 10 μm. (B) Western blot analyses of βC1-YFP and NbNBR1-CFP-HA in co-localization experiments. The blots were probed with anti-βC1 or anti-HA antibodies. The CBB-stained Rubisco large subunit gel was used to show equal sample loadings.
(TIF)

S2 Fig Silencing of *NbNBR1* decreases the accumulation of βC1. (A) Growth phenotypes of the non-silenced (TRV-GFP) and *NbNBR1*-silenced (TRV-NbNBR1) *N*. *benthamiana* plants at 7 dpi. Bar = 2 cm. A fragment (317 nt) of *NbNBR1* gene sequence was amplified and cloned into the TRV RNA2 VIGS vector to produce plasmid RNA2-NbNBR1. Mixed *A*. *tumefaciens* cultures carrying TRV RNA1+RNA2-NbNBR1 (TRV-NbNBR1), or TRV RNA1+RNA2-GFP (TRV-GFP) were individually infiltrated into *N*. *benthamiana* leaves. (B) Quantitative RT-PCR analyses of *NbNBR1* expression in the young leaves of the non-silenced and *NbNBR1*-silenced plants at 7 dpi. Quadruple asterisks indicate a significant statistical difference between the two treatments (****p<0.0001, Student’s *t* test). (C) Growth phenotypes of the Wt, NbNBR1-Cas9-L3, and NbNBR1-Cas9-L4 mutant *N*. *benthamiana* plants. Bar = 2 cm. (D) Symptoms of the PVX- or PVX-βC1-inoculated *NbNBR1*-silenced (TRV-NbNBR1) or non-silenced (TRV-GFP) *N*. *benthamiana* plants at 7 dpi. Bar = 2 cm. (E) Western blot analyses of PVX CP and βC1 accumulations in the assayed plants. The blots were probed with anti-PVX CP or anti-βC1 antibodies. The CBB-stained Rubisco large subunit gel was used to show equal sample loadings.
(TIF)

S3 Fig NbNBR1 could affect the βC1-mediated disease symptom formation and the accumulation of βC1. (A, C) Symptoms of the PVX- or PVX-βC1-inoculated Wt, *NbNBR1*-knockout *N*. *benthamiana* plants at 5 dpi. Bar = 2 cm. (B, D) Western blot analyses of PVX CP and βC1 accumulations in the assayed plants. The blots were probed with anti-PVX CP or anti-βC1 antibodies. The CBB-stained Rubisco large subunit gel was used to show equal sample loadings (B, D).
(TIF)

S4 Fig NbNBR1 does not affect the level of β*C1* mRNA but increases βC1 accumulation through formation of cytoplasmic granules. (A) End point PCR analyses of *βC1* expressions in the *N*. *benthamiana* leaves expressing βC1-YFP and Myc-NbNBR1+βC1-YFP. (B) BiFC assay was performed to investigate the interaction between NbNBR1 and βC1 at different time points. Images were captured under a confocal microscope at 36, 48 and 60 hpi, respectively. Bar = 10 μm. (C, D) Western blot assays were performed to determine the accumulations of NbNBR1 and βC1 using anti-HA antibodies (pEarlyGate201-YN vector used in this study including the HA tag) at various time points. The CBB-stained Rubisco large subunit gel was used to show equal sample loadings (C, D), respectively. (E) A quantification method was used to validate the size of NbNBR1-βC1 complex at different time points. Units: μm.
(TIF)

S5 Fig Overexpression of NbNBR1 promotes PVX-βC1 mediated symptom and enhances the protein level of βC1. (A, C) Symptoms of the PVX- or PVX-βC1-inoculated NbNBR1-overexpressed transgenic *N*. *benthamiana* plants at 5 dpi. Bar = 2 cm. (B, D) Western blot analyses of PVX CP and βC1 accumulations in the assayed plants. The blots were probed with anti-PVX CP or anti-βC1 antibodies. (E) qRT-PCR analyses of *βC1* expressions in TYLCCNV/TYLCCNB infected NbNBR1-YFP-HA transgenic plants. *NbActin* was used as an internal control, and values represent the mean ± standard deviation (SD).
(TIF)

S6 Fig NbNBR1 decreases the ubiquitination level of total protein after TYLCCNV/TYLCCNB infection. (A, B) Western blot analyses of the ubiquitination level of the total protein after TYLCCNV/TYLCCNB infection in both NbNBR1-overexpression transgenic lines (NbNBR1-YFP-HA-L1/L2) (A) and NbNBR1-Cas9 lines (NbNBR1-Cas9-L3/L4) (B). The blots were probed with anti-Ub antibodies. The CBB-stained Rubisco large subunit was used to show equal sample loadings.
(TIF)

S7 Fig Screening the interaction fragment of βC1 with NbNBR1. (A) A summary of interactions between different βC1 mutants and NbNBR1.√stands for interaction while X stands for not. (B) Y2HGold cell cultures transformed with the indicated constructs were first serially diluted (1.0 to 10^−3^) and then grown on the SD-Trp-Leu-His-Ade medium and showed that the first fragment (1–10 aa) of βC1 is response for its interaction with NbNBR1. (C) Western blot analysis of βC1^K4A^-YFP and Myc-NbNBR1 in co-localization experiments. The blots were probed with anti-βC1 or anti-Myc antibodies. The CBB-stained Rubisco large subunit gel was used to show equal sample loadings.
(TIF)

S8 Fig *NbNBR1* expression is up-regulated by βC1. (A) Growth phenotypes of the Wt and the HA-βC1 transgenic *N*. *benthamiana* plants. (B) Expression of *NbNBR1* in the Wt and the HA-βC1 transgenic plants was confirmed through qRT-PCR (*p<0.05, Student’s *t* test). (C, D) End point qPCR assay and qRT-PCR assay were used to validate *βC1* expressions in the *N*. *benthamiana* leaves with indicated combinations. *NbActin* was used as an internal control, and values represent the mean ± SD (B, D). **p<0.01, Student’s *t* test.
(TIF)

S9 Fig Knockout of *NbNBR1* decreases TYLCV-BJ infection. (A, B) qRT-PCR assay and Western blot assay were used to analyze TYLCV-BJ CP accumulations on NbNBR1-knock out lines at 3 dpi. (C) Symptoms of TYLCV-BJ infected NbNBR1-Cas9 lines at 14 dpi. Bar = 2 cm. (D, E) Relative viral CP was analyzed by qRT-PCR assay and Western blot assay. **p<0.01, Student’s *t* test. 25S rRNA was used as an internal control, and values represent the mean ± SD (A, D). The CBB-stained Rubisco large subunit gels were used to show equal sample loadings (B, E).
(TIF)

S10 Fig Overexpression of NbNBR1 promotes TYLCV-BJ infection. (A, B) Total gDNA and protein was extracted from the TYLCV-BJ-infected NbNBR1-YFP-HA transgenic plants at 3 dpi and analyzed for viral CP accumulation through qRT-PCR and Western blot. (*p<0.05, **p<0.01, Student’s *t* test). (C) The TYLCV-BJ-infected NbNBR1-YFP-HA transgenic plants were photographed at 14 dpi. Bar = 2 cm. (D, E) Systemic leaves of TYLCV-BJ infected lines in (C) were harvested and used to validate TYLCV-BJ CP accumulations by qRT-PCR and Western blot. 25S rRNA was used as an internal control, and values represent the mean ± SD (A, D). Double asterisks indicate a significant statistical difference between the two treatments (**p<0.01, Student’s *t* test). The CBB-stained Rubisco large subunit gels were used to show equal sample loadings (B, E).
(TIF)

S11 Fig The TYLCV-BJ encoded V2 protein interacts with NbNBR1. (A) Y2H assay was performed to determine the interaction between NbNBR1 and V2. AD-NbNBR1+BD-NbNBR1 and AD-T+BD-53 were used as positive controls, AD-V2+BD and AD+BD-NbNBR1 were used as negative controls. (B) BiFC assay was performed in the RFP-H2B transgenic *N*. *benthamiana* leaves. At 48 hpi, yellow fluorescence was examined in the cells co-expressing YN-NbNBR1 and YC-V2 or YC-NbNBR1 and YN-V2. Bar = 10 μm.
(TIF)

S1 Table A detailed list of primers used in this study (5’–3’). (DOCX)
