# Supplementary figures and images for: Selective autophagic receptor NbNBR1 prevents NbRFP1-mediated UPS-dependent degradation of βC1 to promote geminivirus infection
Source: PLoS Pathog. 2021 Sep 27;17(9):e1009956. doi: 10.1371/journal.ppat.1009956 (PMC8496818; doi:10.1371/journal.ppat.1009956)

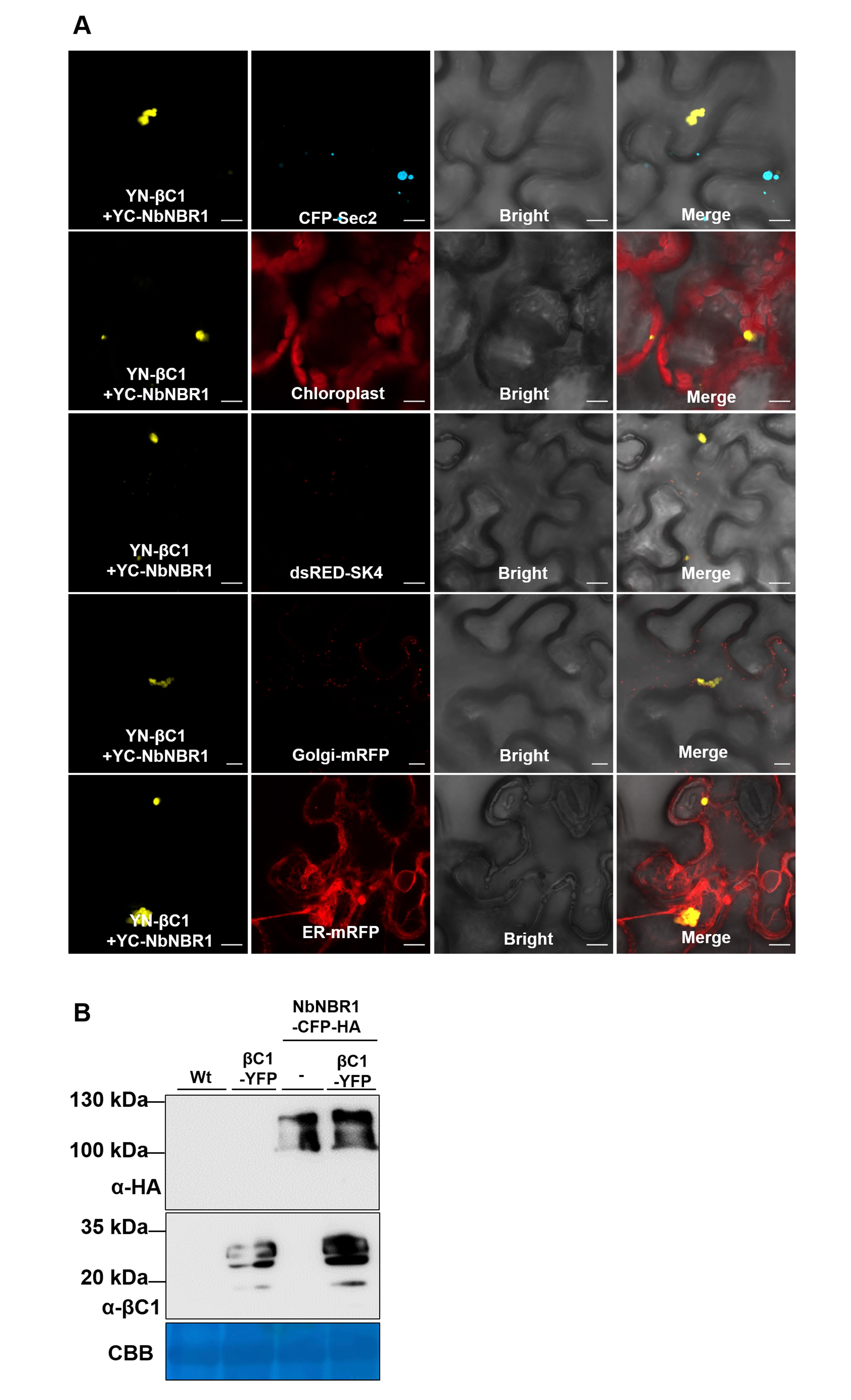

Supplement: S1 Fig — (A) YN-βC1 and YC-NbNBR1 were co-expressed by agrobacteria-mediated infiltration with some reported markers of sub-cellular compartments [Endosome: Sec2 as a marker, chloroplast, peroxisome: SK4 as a marker, Golgi, and endoplamic reticulum (ER)] in N. benthamiana leaves at 48 hpi. Bar = 10 μm. (B) Western blot analyses of βC1-YFP and NbNBR1-CFP-HA in co-localization experiments. The blots were probed with anti-βC1 or anti-HA antibodies. The CBB-stained Rubisco large subunit gel was used to show equal sample loadings. (TIF) [file ppat.1009956.s001.tif]

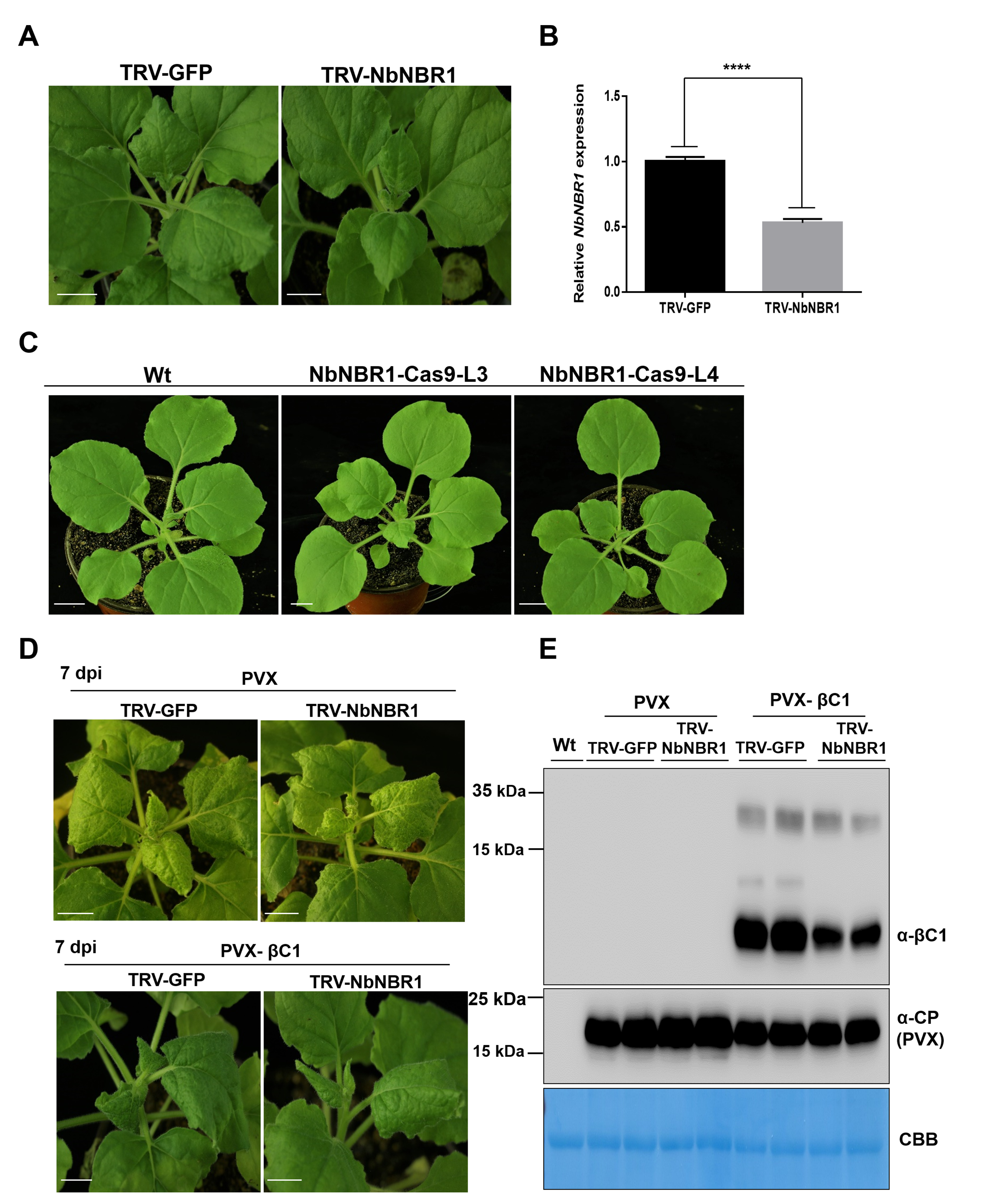

Supplement: S2 Fig — (A) Growth phenotypes of the non-silenced (TRV-GFP) and NbNBR1-silenced (TRV-NbNBR1) N. benthamiana plants at 7 dpi. Bar = 2 cm. A fragment (317 nt) of NbNBR1 gene sequence was amplified and cloned into the TRV RNA2 VIGS vector to produce plasmid RNA2-NbNBR1. Mixed A. tumefaciens cultures carrying TRV RNA1+RNA2-NbNBR1 (TRV-NbNBR1), or TRV RNA1+RNA2-GFP (TRV-GFP) were individually infiltrated into N. benthamiana leaves. (B) Quantitative RT-PCR analyses of NbNBR1 expression in the young leaves of the non-silenced and NbNBR1-silenced plants at 7 dpi. Quadruple asterisks indicate a significant statistical difference between the two treatments (****p<0.0001, Student’s t test). (C) Growth phenotypes of the Wt, NbNBR1-Cas9-L3, and NbNBR1-Cas9-L4 mutant N. benthamiana plants. Bar = 2 cm. (D) Symptoms of the PVX- or PVX-βC1-inoculated NbNBR1-silenced (TRV-NbNBR1) or non-silenced (TRV-GFP) N. benthamiana plants at 7 dpi. Bar = 2 cm. (E) Western blot analyses of PVX CP and βC1 accumulations in the assayed plants. The blots were probed with anti-PVX CP or anti-βC1 antibodies. The CBB-stained Rubisco large subunit gel was used to show equal sample loadings. (TIF) [file ppat.1009956.s002.tif]

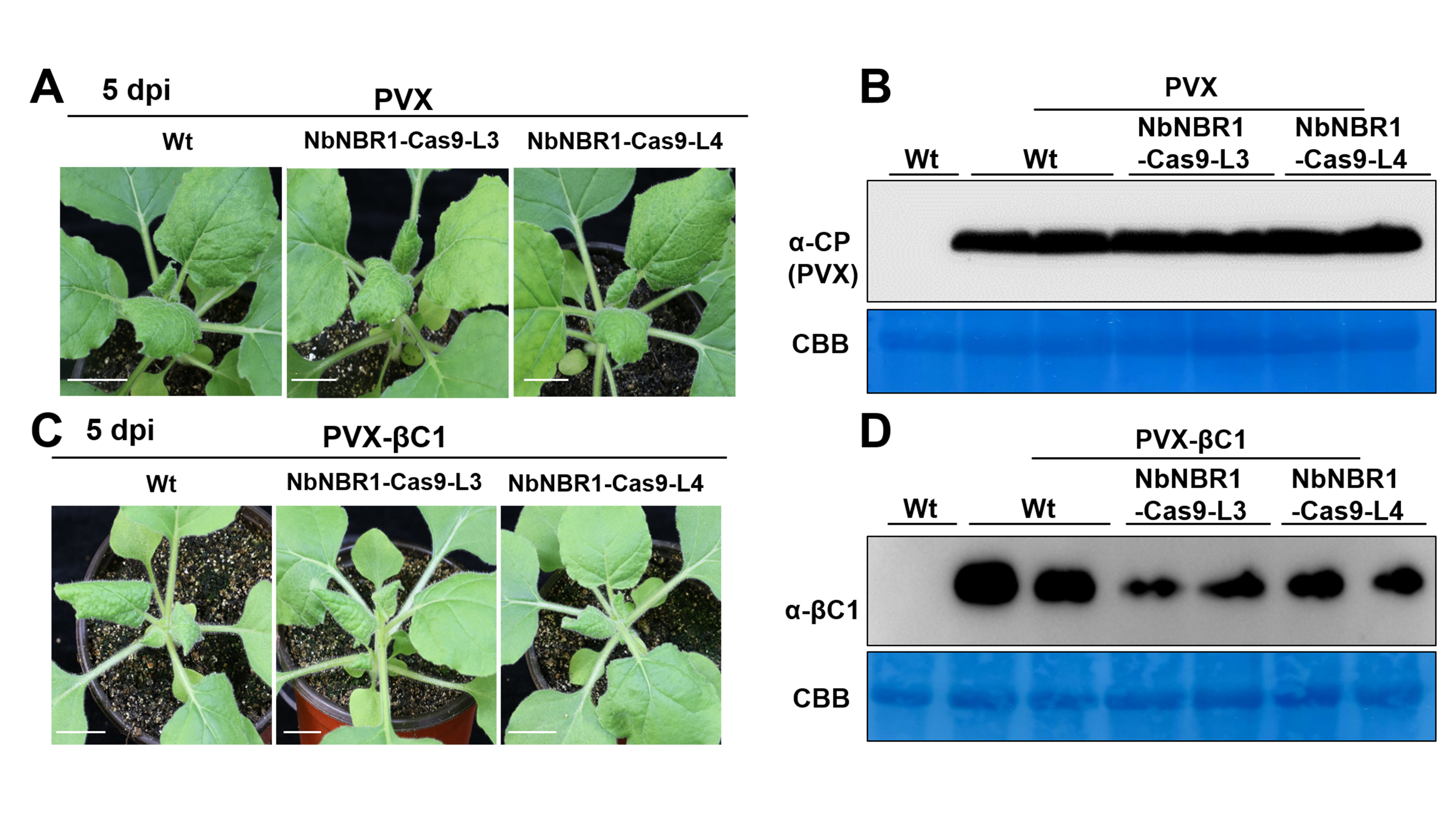

Supplement: S3 Fig — (A, C) Symptoms of the PVX- or PVX-βC1-inoculated Wt, NbNBR1-knockout N. benthamiana plants at 5 dpi. Bar = 2 cm. (B, D) Western blot analyses of PVX CP and βC1 accumulations in the assayed plants. The blots were probed with anti-PVX CP or anti-βC1 antibodies. The CBB-stained Rubisco large subunit gel was used to show equal sample loadings (B, D). (TIF) [file ppat.1009956.s003.tif]

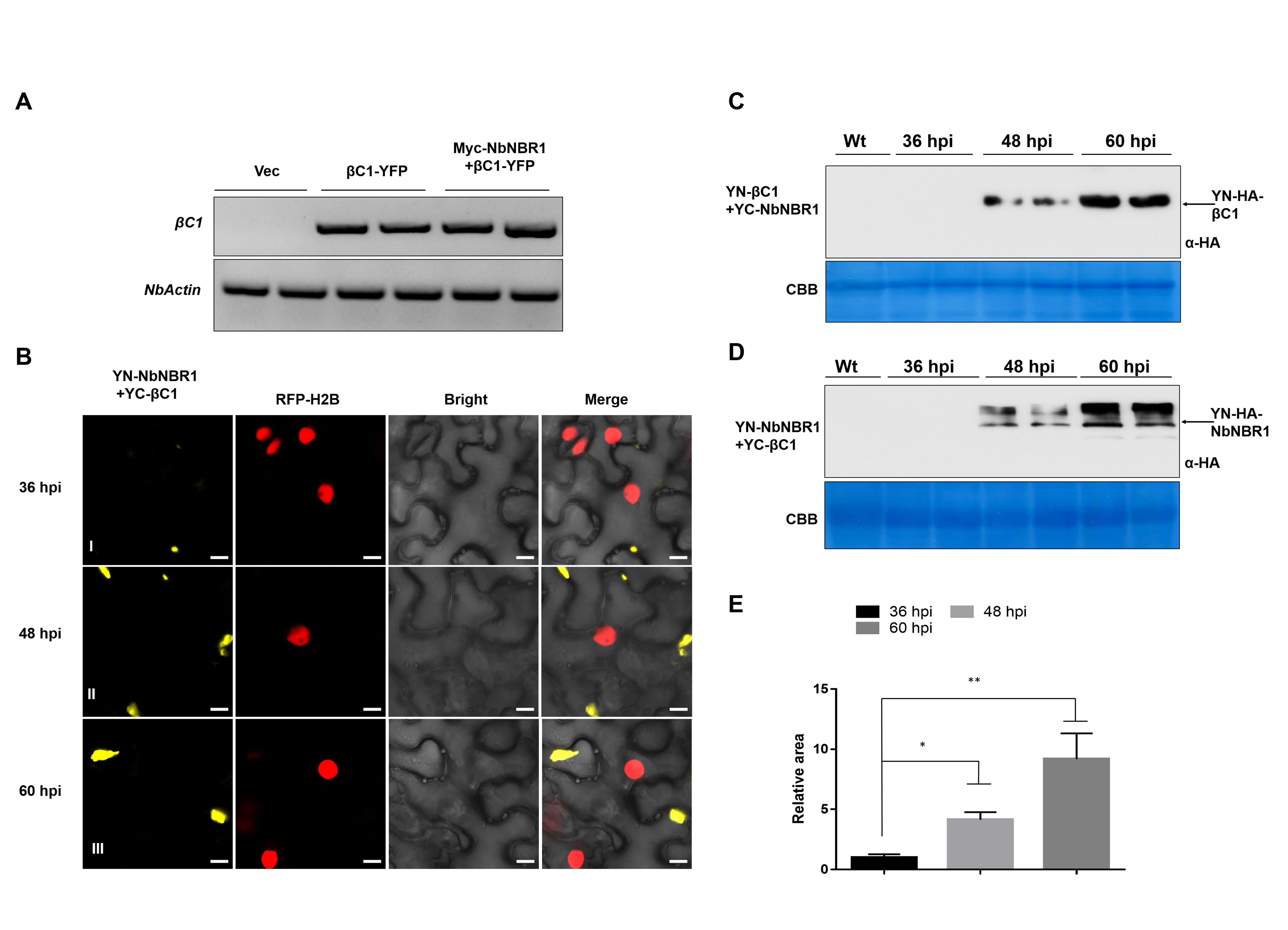

Supplement: S4 Fig — (A) End point PCR analyses of βC1 expressions in the N. benthamiana leaves expressing βC1-YFP and Myc-NbNBR1+βC1-YFP. (B) BiFC assay was performed to investigate the interaction between NbNBR1 and βC1 at different time points. Images were captured under a confocal microscope at 36, 48 and 60 hpi, respectively. Bar = 10 μm. (C, D) Western blot assays were performed to determine the accumulations of NbNBR1 and βC1 using anti-HA antibodies (pEarlyGate201-YN vector used in this study including the HA tag) at various time points. The CBB-stained Rubisco large subunit gel was used to show equal sample loadings (C, D), respectively. (E) A quantification method was used to validate the size of NbNBR1-βC1 complex at different time points. Units: μm. (TIF) [file ppat.1009956.s004.tif]

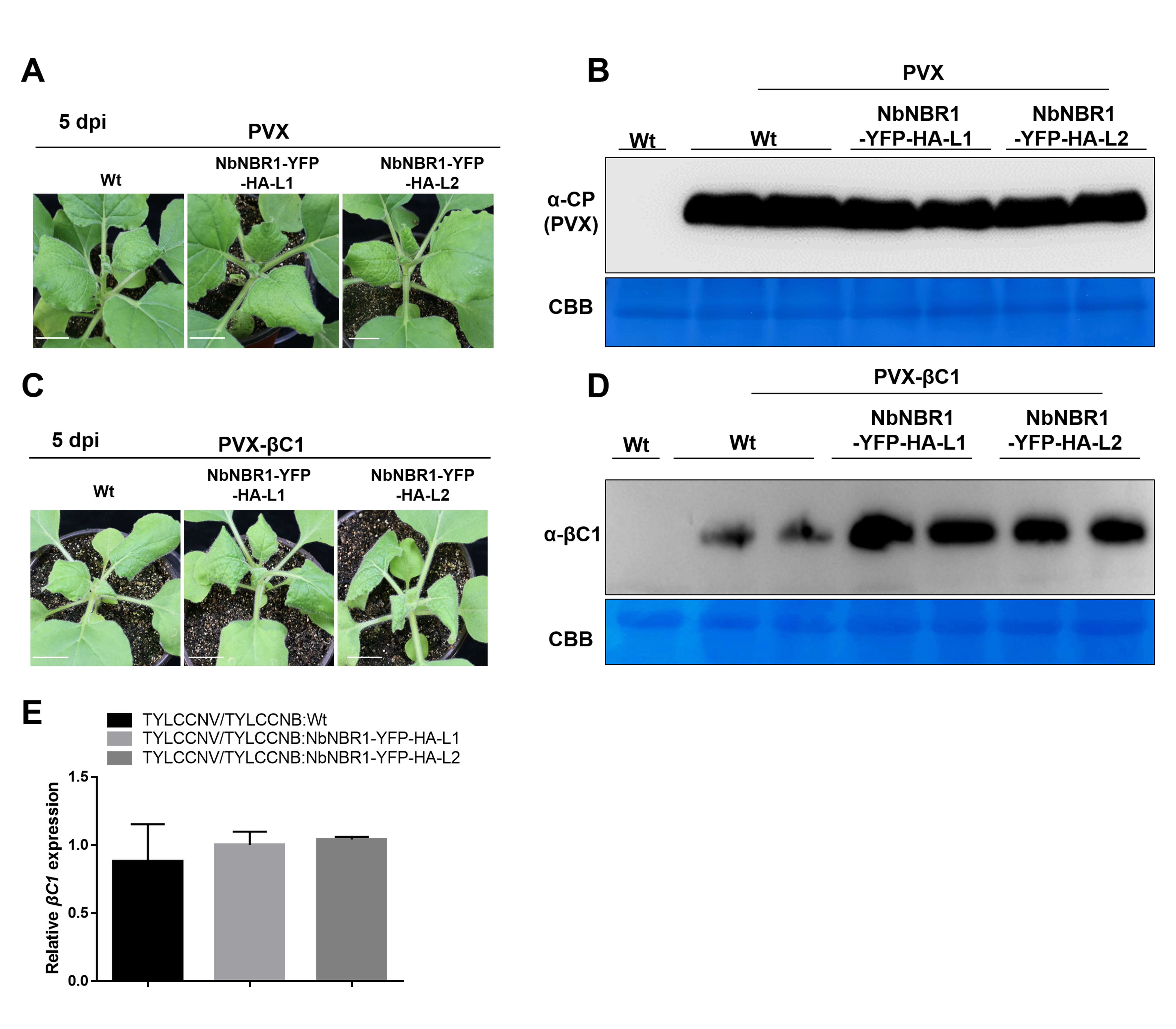

Supplement: S5 Fig — (A, C) Symptoms of the PVX- or PVX-βC1-inoculated NbNBR1-overexpressed transgenic N. benthamiana plants at 5 dpi. Bar = 2 cm. (B, D) Western blot analyses of PVX CP and βC1 accumulations in the assayed plants. The blots were probed with anti-PVX CP or anti-βC1 antibodies. (E) qRT-PCR analyses of βC1 expressions in TYLCCNV/TYLCCNB infected NbNBR1-YFP-HA transgenic plants. NbActin was used as an internal control, and values represent the mean ± standard deviation (SD). (TIF) [file ppat.1009956.s005.tif]

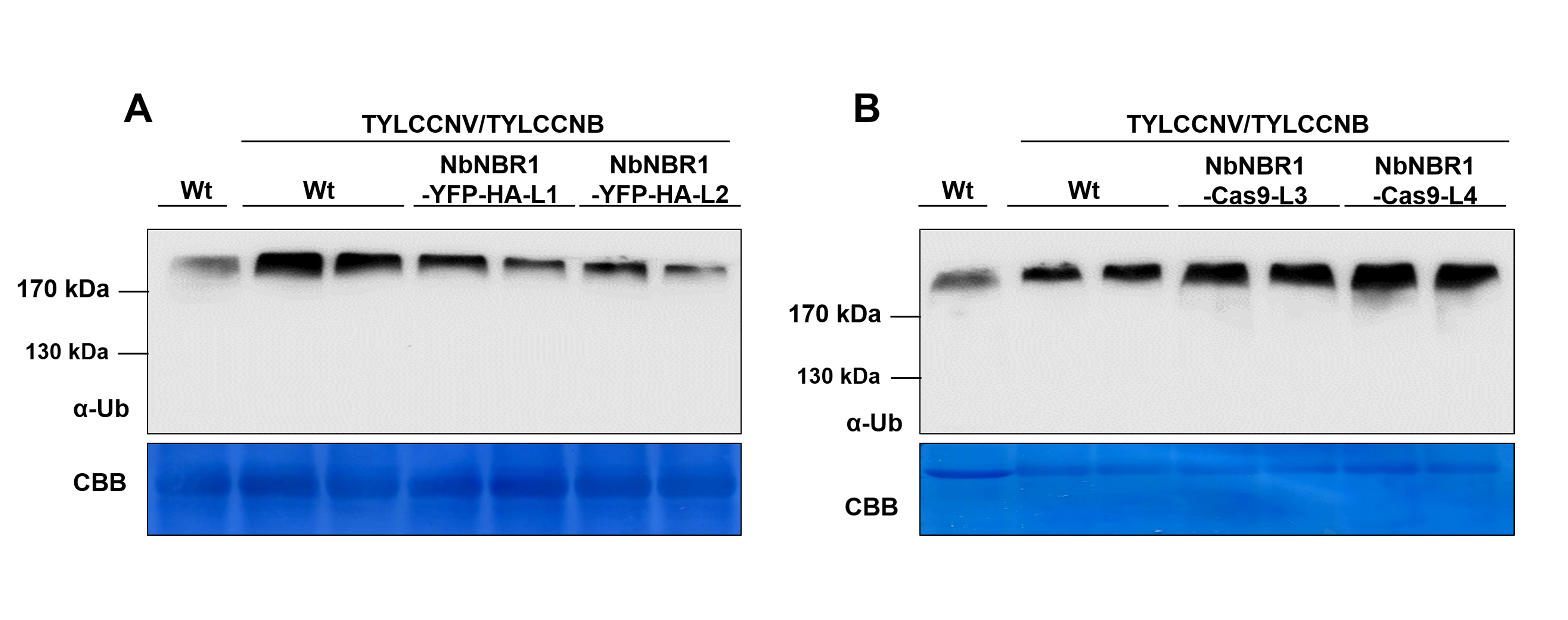

Supplement: S6 Fig — (A, B) Western blot analyses of the ubiquitination level of the total protein after TYLCCNV/TYLCCNB infection in both NbNBR1-overexpression transgenic lines (NbNBR1-YFP-HA-L1/L2) (A) and NbNBR1-Cas9 lines (NbNBR1-Cas9-L3/L4) (B). The blots were probed with anti-Ub antibodies. The CBB-stained Rubisco large subunit was used to show equal sample loadings. (TIF) [file ppat.1009956.s006.tif]

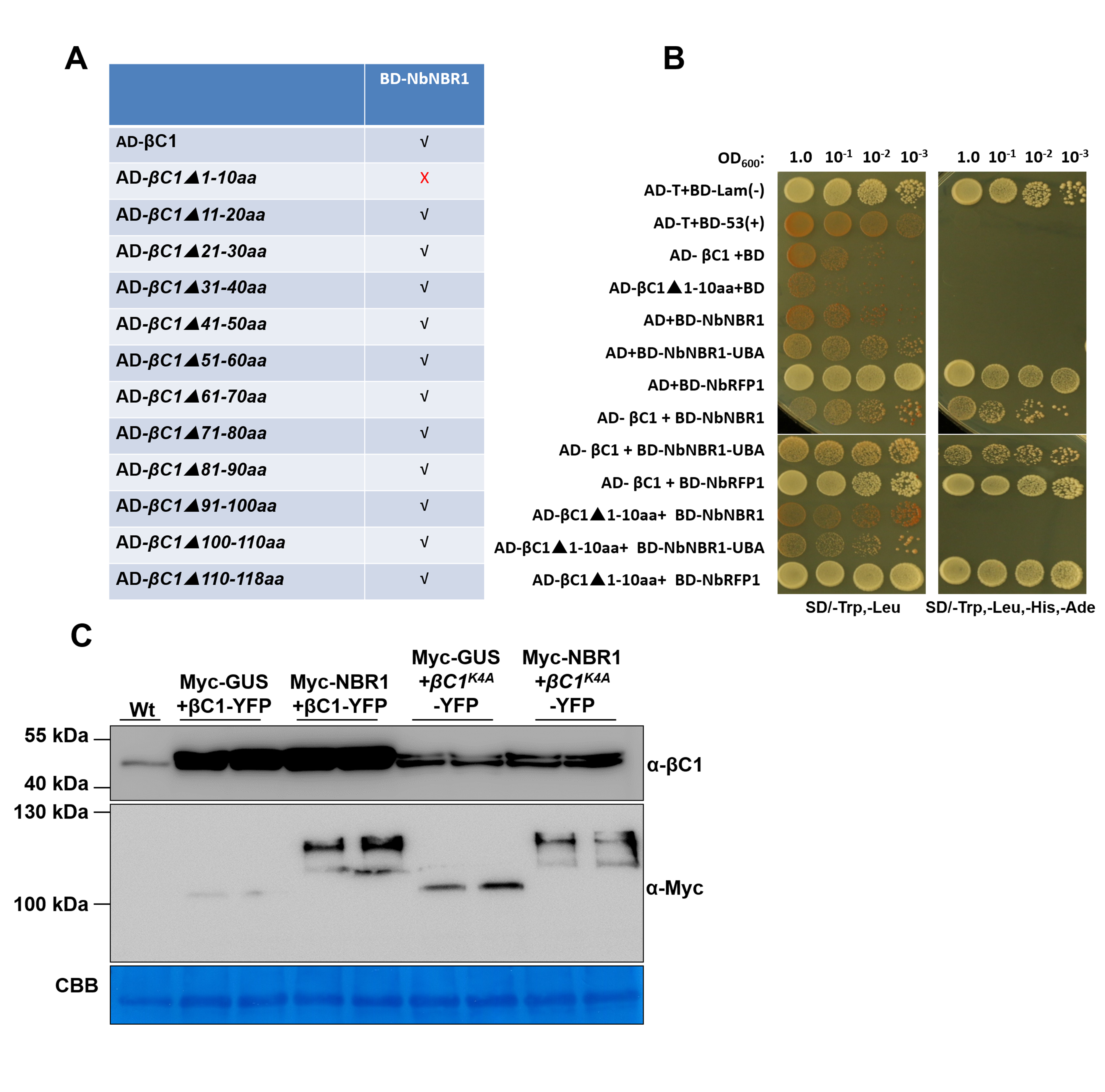

Supplement: S7 Fig — (A) A summary of interactions between different βC1 mutants and NbNBR1.√stands for interaction while X stands for not. (B) Y2HGold cell cultures transformed with the indicated constructs were first serially diluted (1.0 to 10−3) and then grown on the SD-Trp-Leu-His-Ade medium and showed that the first fragment (1–10 aa) of βC1 is response for its interaction with NbNBR1. (C) Western blot analysis of βC1K4A-YFP and Myc-NbNBR1 in co-localization experiments. The blots were probed with anti-βC1 or anti-Myc antibodies. The CBB-stained Rubisco large subunit gel was used to show equal sample loadings. (TIF) [file ppat.1009956.s007.tif]

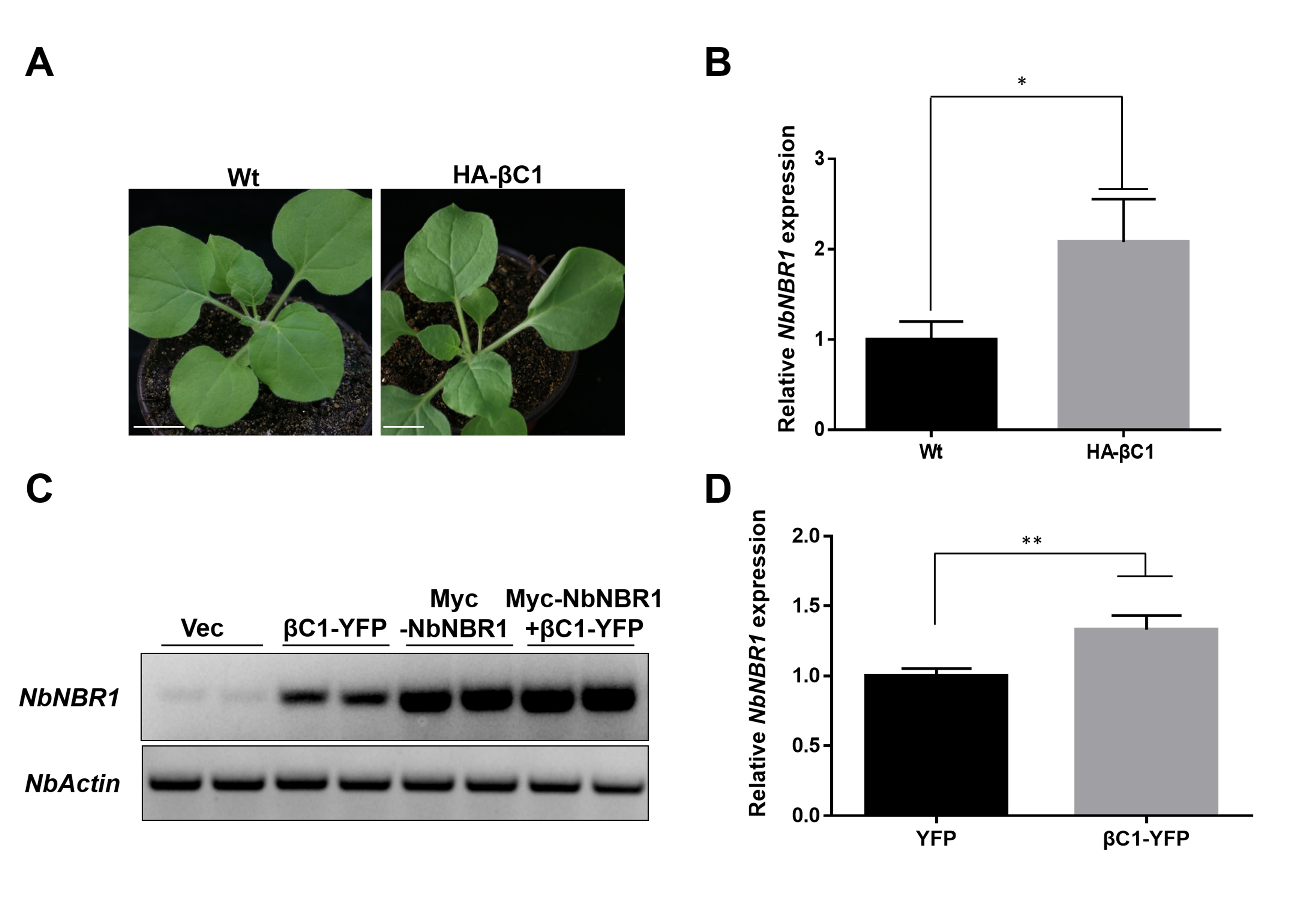

Supplement: S8 Fig — (A) Growth phenotypes of the Wt and the HA-βC1 transgenic N. benthamiana plants. (B) Expression of NbNBR1 in the Wt and the HA-βC1 transgenic plants was confirmed through qRT-PCR (*p<0.05, Student’s t test). (C, D) End point qPCR assay and qRT-PCR assay were used to validate βC1 expressions in the N. benthamiana leaves with indicated combinations. NbActin was used as an internal control, and values represent the mean ± SD (B, D). **p<0.01, Student’s t test. (TIF) [file ppat.1009956.s008.tif]

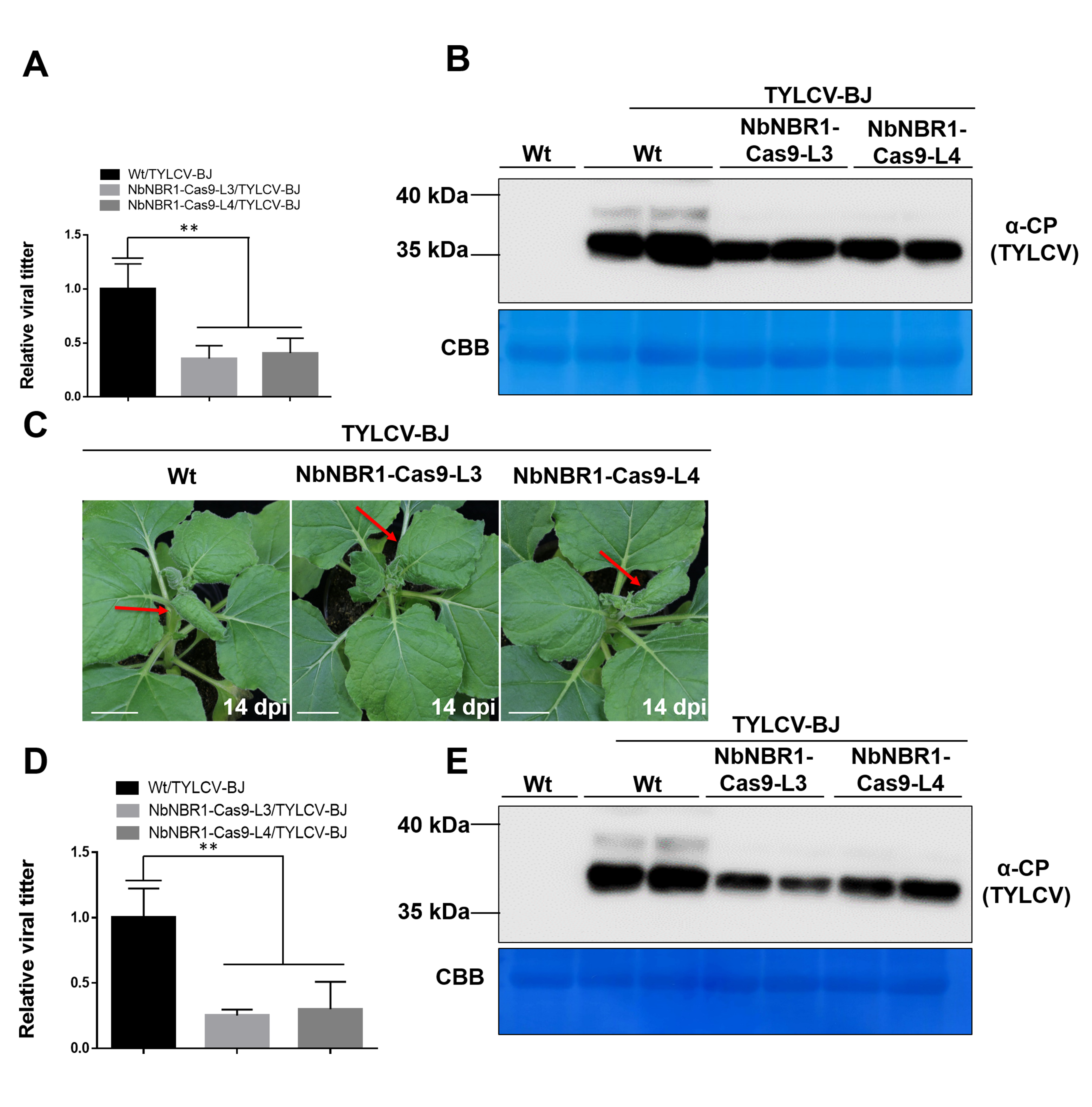

Supplement: S9 Fig — (A, B) qRT-PCR assay and Western blot assay were used to analyze TYLCV-BJ CP accumulations on NbNBR1-knock out lines at 3 dpi. (C) Symptoms of TYLCV-BJ infected NbNBR1-Cas9 lines at 14 dpi. Bar = 2 cm. (D, E) Relative viral CP was analyzed by qRT-PCR assay and Western blot assay. **p<0.01, Student’s t test. 25S rRNA was used as an internal control, and values represent the mean ± SD (A, D). The CBB-stained Rubisco large subunit gels were used to show equal sample loadings (B, E). (TIF) [file ppat.1009956.s009.tif]

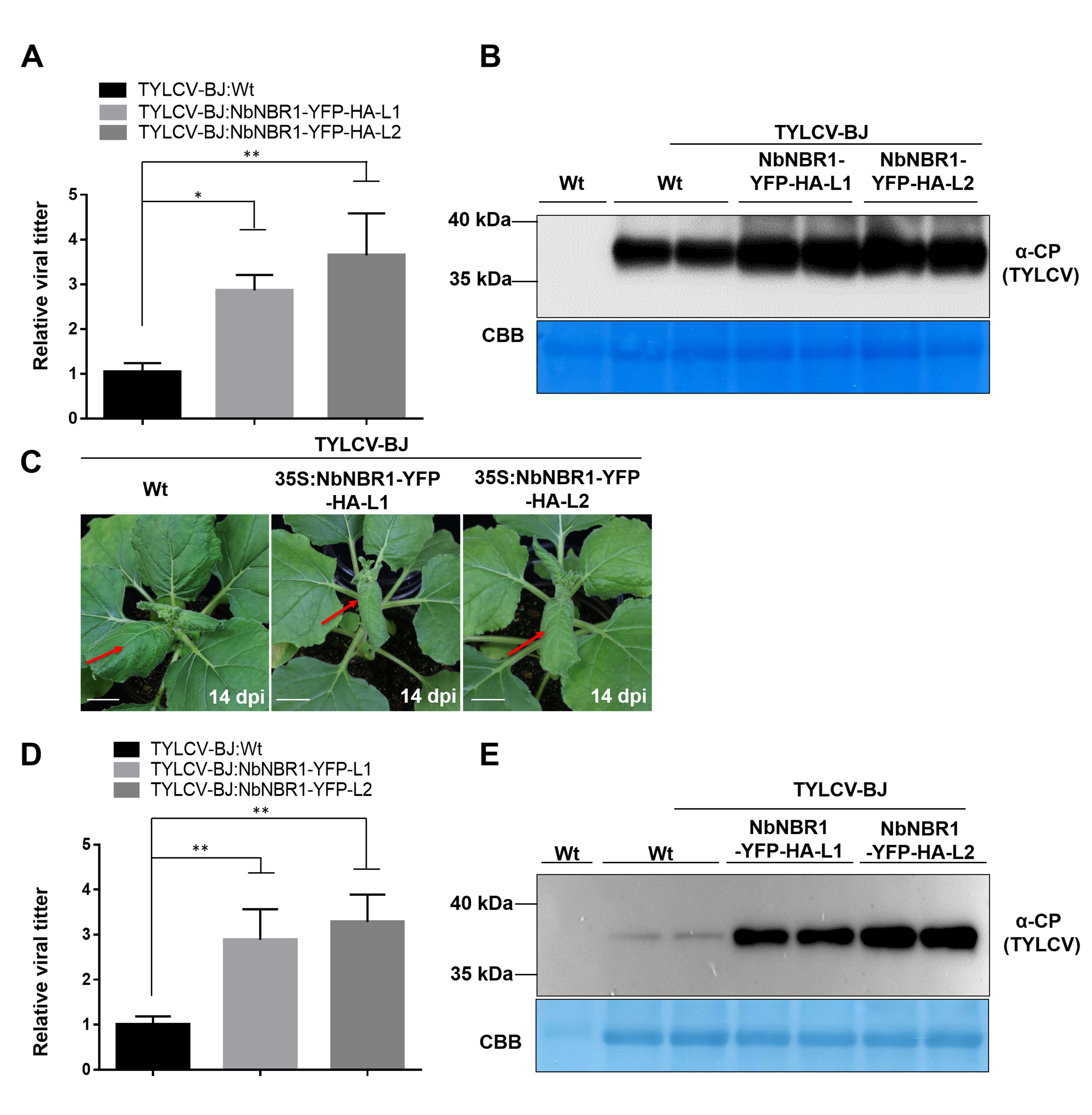

Supplement: S10 Fig — (A, B) Total gDNA and protein was extracted from the TYLCV-BJ-infected NbNBR1-YFP-HA transgenic plants at 3 dpi and analyzed for viral CP accumulation through qRT-PCR and Western blot. (*p<0.05, **p<0.01, Student’s t test). (C) The TYLCV-BJ-infected NbNBR1-YFP-HA transgenic plants were photographed at 14 dpi. Bar = 2 cm. (D, E) Systemic leaves of TYLCV-BJ infected lines in (C) were harvested and used to validate TYLCV-BJ CP accumulations by qRT-PCR and Western blot. 25S rRNA was used as an internal control, and values represent the mean ± SD (A, D). Double asterisks indicate a significant statistical difference between the two treatments (**p<0.01, Student’s t test). The CBB-stained Rubisco large subunit gels were used to show equal sample loadings (B, E). (TIF) [file ppat.1009956.s010.tif]

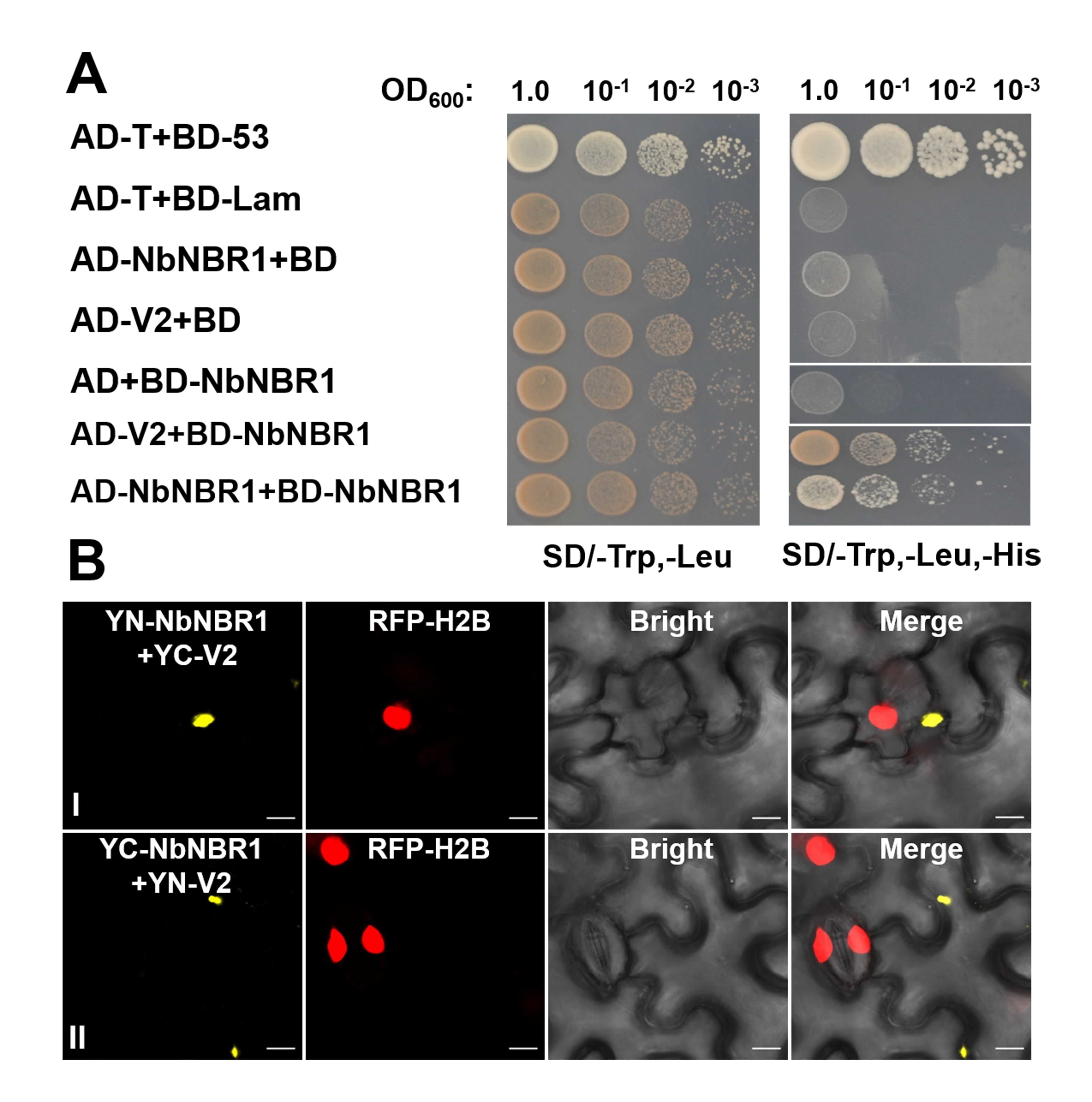

Supplement: S11 Fig — (A) Y2H assay was performed to determine the interaction between NbNBR1 and V2. AD-NbNBR1+BD-NbNBR1 and AD-T+BD-53 were used as positive controls, AD-V2+BD and AD+BD-NbNBR1 were used as negative controls. (B) BiFC assay was performed in the RFP-H2B transgenic N. benthamiana leaves. At 48 hpi, yellow fluorescence was examined in the cells co-expressing YN-NbNBR1 and YC-V2 or YC-NbNBR1 and YN-V2. Bar = 10 μm. (TIF) [file ppat.1009956.s011.tif]
