# Supplementary material for: Selective autophagic receptor NbNBR1 prevents NbRFP1-mediated UPS-dependent degradation of βC1 to promote geminivirus infection
Source: PLoS Pathog. 2021 Sep 27;17(9):e1009956. doi: 10.1371/journal.ppat.1009956 (PMC8496818; doi:10.1371/journal.ppat.1009956)
Supplement: S1 Table — (DOCX) [file ppat.1009956.s012.docx]

**S1 Table. A detailed list of primers used in this study (5’–3’).**

| **AD/BD-NbNBR1 construction** | **Primer sequences** | **Descriptions** |
| --- | --- | --- |
| AD-NbNBR1-NdeI-F | GTACCAGATTACGCTCATATG ATGGCTATGGAGTCTGCTATTG | Primers for AD-NbNBR1 construction |
| AD-NbNBR1-EcoRI-R | ATGCCCACCCGGGTGGAATTC CTACTGCTCTCCAGCAATAAGA |  |
| BD-NbNBR1-NdeI-F | TCAGAGGAGGACCTGCATATG ATGGCTATGGAGTCTGCTATTG | Primers for BD-NbNBR1 construction |
| BD-NbNBR1-EcoRI-R | TCGACGGATCCCCGGGAATTC CTACTGCTCTCCAGCAATAAGA |  |
| **TRV-based VIGS** |  |  |
| TRV-NbNBR1-EcoRI-F | cggaattc ATGGGACTGGGACTATTTTCCA | Primers for TRV-NbNBR1 construction |
| TRV-NbNBR1-SmaI-R | cccccggg AAACGGCTGTCGAACTTTGGCC |  |
| NbNBR1-qp-1F | CCTCCTCTTGAGGTCCCATATAA | Primers for NbNBR1 mRNA test |
| NbNBR1-qp-1R | CACAACCATCACAACGAACAC |  |
| **Viral accumulation test by qPCR** |  |  |
| q-25S-rRNA-F | ATAACCGCATCAGGTCTCCA | Primers for qPCR reference gene |
| q-25S-rRNA-R | CCGAAGTTACGGATCCATTT |  |
| Y10/CP(118-141)-F： | AGAAGACAAATGTGGTCCAACAGG | Primers for TYLCCNV test |
| Y10/CP(448-474)-R： | GCAATTAAAGACTTGTTGGAAATCCAT |  |
| Y10β/qRT-PCR-F： | ATGACTATCAAATACAACAACATGAAGGGT | Primers for TYLCCNB test |
| Y10β/qRT-PCR-R： | TCATCCCCTACATCTATATCTTCTACTGG |  |
| **NbNBR1-Cas9 transgene plants** |  |  |
| NbNBR1-spacer1-F | TGATTGCACAGCACTGAATGATGCA | Primers for NbNBR1-Cas9 construction |
| NbNBR1-spacer1-R | AAACTGCATCATTCAGTGCTGTGCA |  |
| BGK-Cas9-F | CAGCCAGGAAGAGTTCTACAAG | Primers for detecting NbNBR1-Cas9 lines |
| BGK-Cas9-R | CATTCCCTCGGTCACGTATTT |  |
| NbNBR1-spacer1-detect-F | GAATTCCAGCGTTTCTGATGCT | Primers for detecting NbNBR1-Cas9 lines |
| NbNBR1-spacer1-detect-R | GAAGGCAAGAGGCTTTCCACCA |  |
| **NbRFP1 mRNA expression level test** |  |  |
| NbRFP1-qRT-F | CTGCATCACATCGGGCAGAATTTGA | Primers for NbRFP1 mRNA test |
| NbRFP1-qRT-R | CTCTGTAACGAGATAGAACTTCCTC |  |
| **Southern blot** |  |  |
| Y10A-PROBE-F | GAAGAACCATACAAACACCGT | Primers for TYLCCNV Southern blot probe |
| Y10A-PROBE-R | TTCCTGAGGAACCACGACGT |  |
| Y10β-PROBE-F | GTAGGTACCACTACGCTACGCAGCAGCC | Primers for TYLCCNB Southern blot probe |
| Y10β-PROBE-R | AGTGGTACCTACCCTCCCAGGGGTACAC |  |
| **βC1^K4A^ mutant and TYLCCNBK4A construction** |  |  |
| βC1^K4A^ -F | AAGCAGGCTTCATGACTATCGCATACAACAACATGAAGGG | Primers for βC1^K4A^ mutant construction |
| βC1^K4A^ -R | CCCTTCATGTTGTTGTATGCGATAGTCATGAAGCCTGCTT |  |
| INFU-DNAB-SalI-F | CTTGCATGCCTGCAGGTCGAC TACCCTCCCAGGGGTACAC | Primers for TYLCCNB^K4A^ mutant construction |
| INFU-DNAB-KpnI-R | TAAGAATTCGAGCTCGGTACC AAACCACTACGCTACGCAGCAGCC |  |
| DNAB-K4A-F | gttgttgtaCGTgatagtcatgtttatttgttgtgg |  |
| DNAB-K4A-R | atgactatcGCAtacaacaacatgaagggtttggag |  |
| T-DNAB-KpnI-F | GGTACCTACCCTCCCAGGGGTACAC |  |
| T-DNAB-EcoRI-R | GAATTCGAAACCACTACGCTACGCAGCAGCC |  |
| pGD-βC1^K4A^-HA-XholI-INF | cgatagggatcctggctcgag ATGACTATCGCATACAACAACA | Primers for βC1K4A-HA mutant construction |
| pGD-βC1^K4A^-HA-SacI-INR | AACATCGTATGGGTAgagctc TACATCTGAATTTGTAAATACA |  |
| PVX-βC1^K4^-ClaI-F | tcagcaccagctagcatcgat ATGACTATCGCATACAACAACA | Primers for PVX-βC1K4A mutant construction |
| PVX-βC1^K4^-SalI-R | aaccgttcatcggcggtcgac TACATCTGAATTTGTAAATACA |  |
| **Y3H** |  |  |
| pBRIGE-NbRFP1-SalI-F | AATTCCCGGGGATCCGTCGACCT atgggtggaaagagttcaagag | Primers for pBRIDGE-NbRFP1 construction |
| pBRIGE -NbRFP1-SalI-R | TTAGCTTGGCTGCAGGTCGAC gtagagctttattcgggttaga |  |
| pBRIGE -NbNBR1-NotI-F | AAGAAGAGAAAGGTGGCGGCCGCA ATGGCCATGGAGTCTGCTATT | Primers for pBRIDGE-NbNBR1 construction |
| pBRIGE -NbNBR1-BGII-R | GGGAGATCAGCCCGAAGATCT CTGCTCTCCAGCAATAAGATC |  |
| pBRIGE -MBP-BamHI-F | GAAGAGAAAGGTGGCGGCCGCAatgaaaatcgaagaag | Primers for pBRIDGE-MBP construction |
| pBRIGE -MBP-PstI-R | GGGAGATCAGCCCGAAGATCTagtctgcgcgtctttc |  |
| **NbRFP1 construction** |  |  |
| gw-221-NbRFP1-F | GGGGACAAGTTTGTACAAAAAAGCAGGCTTC atgggtggaaagagttcaagag | Primers for 221-NbRFP1 construction |
| gw-221-NbRFP1-R | GGGGACCACTTTGTACAAGAAAGCTGGGTC gtagagctttattcgggttaga |  |
